# Inhibition of mitosomal alternative oxidase causes lifecycle arrest of early-stage *Trachipleistophora hominis* meronts during intracellular infection of mammalian cells

**DOI:** 10.1371/journal.ppat.1011024

**Published:** 2022-12-20

**Authors:** Kacper M. Sendra, Andrew K. Watson, Ekaterina Kozhevnikova, Anthony L. Moore, T. Martin Embley, Robert P. Hirt

**Affiliations:** 1 Biosciences Institute, Newcastle University, Newcastle upon Tyne, United Kingdom; 2 Department of Biochemistry and Biomedicine, School of Life Sciences, University of Sussex, Falmer, Brighton, United Kingdom; Deakin University, Australia School of Medicine, AUSTRALIA

## Abstract

Mitosomes are highly reduced forms of mitochondria which have lost two of the ‘defining’ features of the canonical organelle, the mitochondrial genome, and the capacity to generate energy in the form of ATP. Mitosomes are found in anaerobic protists and obligate parasites and, in most of the studied organisms, have a conserved function in the biosynthesis of iron-sulfur clusters (ISC) that are indispensable cofactors of many essential proteins. The genomes of some mitosome-bearing human pathogenic Microsporidia encode homologues of an alternative oxidase (AOX). This mitochondrial terminal respiratory oxidase is absent from the human host, and hence is a potential target for the development of new antimicrobial agents. Here we present experimental evidence for the mitosomal localization of AOX in the microsporidian *Trachipleistophora hominis* and demonstrate that it has an important role during the parasite’s life cycle progression. Using a recently published methodology for synchronising *T*. *hominis* infection of mammalian cell lines, we demonstrated specific inhibition of *T*. *hominis* early meront growth and replication by an AOX inhibitor colletochlorin B. Treatment of *T*. *hominis-*infected host cells with the drug also inhibited re-infection by newly formed dispersive spores. Addition of the drug during the later stages of the parasite life cycle, when our methods suggest that AOX is not actively produced and *T*. *hominis* mitosomes are mainly active in Fe/S cluster biosynthesis, had no inhibitory effects on the parasites. Control experiments with the AOX-deficient microsporidian species *Encephalitozoon cuniculi*, further demonstrated the specificity of inhibition by the drug. Using the same methodology, we demonstrate effects of two clinically used anti-microsporidian drugs albendazole and fumagillin on the cell biology and life cycle progression of *T*. *hominis* infecting mammalian host cells. In summary, our results reveal that *T*. *hominis* mitosomes have an active role to play in the progression of the parasite life cycle as well as an important role in the biosynthesis of essential Fe/S clusters. Our work also demonstrates that *T*. *hominis* is a useful model for testing the efficacy of therapeutic agents and for studying the physiology and cell biology of microsporidian parasites growing inside infected mammalian cells.

## Author summary

Microsporidia are increasingly appreciated as ubiquitous pathogens with ecological, veterinary and medical importance. Here we provide detailed protocol for establishing a synchronised infection of microsporidian parasites *T*. *hominis* and *E*. *cuniculi* inside host RK13 cell lines. We demonstrate how this protocol in combination with molecular and cell biology techniques can be utilised to test the effects of anti-microsporidian drugs, and specific protein inhibitors on the parasite’s cell biology and infection cycle progression. Using these techniques with bespoke specific antibodies against *T*. *hominis* alternative respiration (AR) components—alternative oxidase (AOX) and glycerol-3-phosphate dehydrogenase (mtG3PDH)—we demonstrate their life cycle stage-dependent mitosomal localisation, and in combination with the specific AOX-inhibitor colletochlorin B we show an essential function of the mitosomal AOX during an early phase of the *T*. *hominis* infection cycle. Using bioinformatics and specific inhibitors we show that the role of the microsporidian minimal mitochondria in AR was subsequently lost in the course of further reductive evolution in some lineages including that of *E*. *cuniculi*. Additional RNAseq across *T*. *hominis* life cycle and experiments using antibodies against the mitosomal iron-sulphur clusters biosynthesis (ISC) protein NFS indicated that unlike the phylogenetically-restricted AR, the conserved ISC function of the mitosome is active during *T*. *hominis* proliferation.

## Introduction

Obligate intracellular Microsporidia are a large group of parasites of emerging clinical and agricultural significance [1]. Due to their extreme dependency on the host, Microsporidia can only survive in the environment outside of a host cell in the form of a resistant dispersive spore [2]. The microsporidian spore is surrounded by two layers of a spore wall, and is equipped with a specialized needle-like infection apparatus, called a polar tube, used to inject the spore contents into the host cell [2]. After invading the host cell, Microsporidia can utilize members of two different types of major facilitator superfamily nucleotide transport proteins to steal nucleotides, including ATP and GTP, from the infected host cell [3–6]. These surface located transporters, are among the key adaptations enabling Microsporidia to parasitize a taxonomically broad range of eukaryotic hosts, including immunocompromised humans [1], and economically important fish, crustaceans, and honeybees [7]. The adoption of energy and nucleotide parasitism has allowed the loss by microsporidians of the ability to make their own nucleotides *de novo* and to make energy using the mitochondrial electron transport chain [5,8,9]. The loss of the electron transport chain in turn has allowed Microsporidia to lose the mitochondrial genome [3,10–12]. The biosynthesis of essential iron-sulfur clusters (ISC) using nuclear encoded proteins, now appears to be the only conserved function of the minimal microsporidian organelle, which are now commonly called mitosomes [11–13].

While all Microsporidia studied in detail appear to have lost the ability to make ATP using their mitosomes, some species including *Paranosema (Antonospora) locustae* and *Trachipleistophora hominis*, have retained the capacity to make cytosolic ATP using glycolysis [8,14–16]. *Trachipleistophora hominis*, the main focus of the current study, was originally isolated from the muscles of an infected HIV/AIDS patient [17]. Consistent with the ability of the parasites to steal ATP while living inside host cells [3–5], glycolytic enzymes were detected mainly in the spores of *Paranosema (Antonospora) locustae* [16] and *Trachipleistophora hominis* [15], where they are thought to be used to provide ATP and reducing power for spore survival and germination.

In glycolysis, ADP is phosphorylated to ATP, and NAD^+^ is reduced to NADH, which must eventually be re-oxidized back to NAD^+^ in order to maintain the capacity for ATP-generation by the pathway. In *Paranosema (Antonospora) locustae* and *T*. *hominis*, the regeneration of NAD^+^ for glycolysis can be carried out by an alternative respiratory (AR) pathway located in their cytosol and mitosomes [16,18]. The microsporidian AR pathway consists of homologues of a single cytosolic enzyme (cytosolic glycerol-3-phosphate dehydrogenase, cytG3PDH) and two mitochondrial enzymes (mitochondrial glycerol-3-phosphate dehydrogenase, mtG3PDH; and alternative oxidase, AOX). Acting together these enzymes are thought to be able to regenerate the NAD^+^ required during glycolysis by the transfer of electrons and protons from cytosolic NADH to molecular oxygen inside mitosomes [18]. AOX, the enzyme catalysing the cyanide-resistant transfer of electrons from ubiquinol to oxygen [19,20], can be found in the mitochondria of other human pathogens including parasitic fungi [21,22] and protists [18,23,24], but is not encoded in the human genome. This has resulted in AOX becoming a novel target for the development of new antimicrobial agents [19,20], and inhibitors of AOX have been used in the treatment of animals infected with *Trypanosoma brucei*, a causative agent of African sleeping sickness [25,26]. Bloodstream forms of *T*. *brucei* rely on AOX to oxidize NADH generated by the glycolytic pathway [27]. Since AOX is the only mitosomal terminal oxidase encoded in the genome of *T*. *hominis* [15] and some other Microsporidia [18], it represents a potential target for a group for which there are relatively few effective therapeutic strategies [28].

Here, we used a combination of molecular cell biology and transcriptomics to investigate the importance of AOX in *T*. *hominis*. We used specific antibodies to determine the intracellular localization of the *T*. *hominis* respiratory enzymes (AOX and mtG3PDH), and to study their expression across a synchronised time course of mammalian cell infection. To investigate the role of mitosomal respiration in the infection process, we investigated the effects of specifically inhibiting AOX in *T*. *hominis* and the AOX-deficient microsporidian *Encephalitozoon cuniculi* using a known inhibitor of the enzyme. In parallel, we also investigated the effects of two clinically used anti-microsporidian agents (albendazole and fumagillin) on the growth and replication of *T*. *hominis*. Our results demonstrate that the *T*. *hominis* mitosomal AOX has an important role in the early meront stages and progression of *T*. *hominis* infection.

## Results and discussion

### AOX and mtG3PDH are localized inside *T*. *hominis* mitosomes

**Fig 1:**
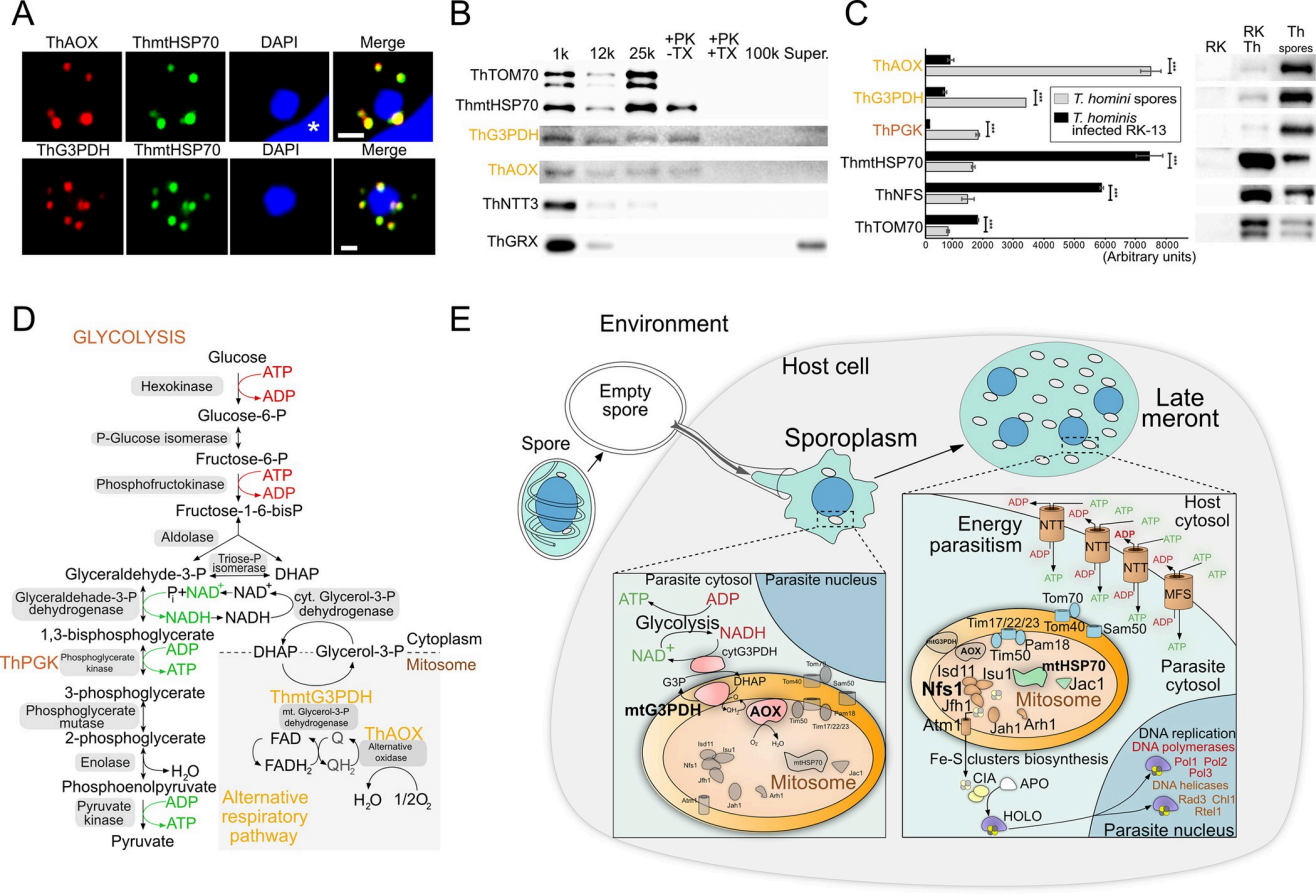
The components of the microsporidian alternative respiratory pathway localise inside *T*. *hominis* mitosome and are enriched in *T*. *hominis* spores rather than intracellular stages from infected host rabbit kidney cells. **(A)** Confocal immunfluorescence images of single *T*. *hominis* cells inside the infected host RK13 cells. Monolayer of *T*. *hominis* infected host cells was fixed with methanol-acetone and double labelled with the affinity purified polyclonal rabbit antibodies against *T*. *hominis* AOX (red, top panel) or *T*. *hominis* mtG3PDH (red, bottom panel) and polyclonal rat antisera raised against *T*. *hominis* mtHSP70 (green). Nuclei of the parasite and the host (asterisk) were labelled with DAPI (blue), scale bars correspond to 1 μm. (**B)** Fractionation by differential ultracentrifugation was performed on lysates of *T*. *hominis* infected RK13 cells lysed with a glass homogeniser. Western blots of protein fractions were probed with the affinity purified antibodies against *T*. *hominis* AOX and mtG3PDH, as well as polyclonal sera against characterized *T*. *hominis* proteins that localize to: outer mitosomal membrane (ThTOM70) [13], mitosomal matrix (mtHSP70) [10], cell membrane (ThNTT3) [5], and cytosol (ThGRX) [13]. Mitosome-enriched 25,000g (25k) fraction was treated with proteinase K (+PK–TX), or proteinase K and Triton X-100 (+PK +TX). Supernatant fraction (super.) is a supernatant after the final 100,000g (100k) centrifugation. (**C)** Analyses of the intensities of the specific bands detected in protein extracts from purified *T*. *hominis* spores (Th spores), *T*. *hominis* infected RK13 cells (RKTh) and non-infected RK13 (RK) cells control. The western blots were probed with affinity purified antibodies against ThAOX or ThmtG3PDH, and polyclonal sera against ThPGK, ThmtHSP70, ThNFS, and ThTOM70. The intensities of the detected bands were measured with FIJI [31]. Significantly different values were indicated (T-test; ***, P ≤ 0.001). Error bars represent standard deviation of intensities values measured in three replicate western blotting experiments. 10 μg (BCA assay) of each protein extract were loaded onto gels (S3 Fig). **(D)** Hypothetical model of the ATP producing pathway consisting of glycolysis and mitosomal alternative respiratory pathway based on bioinformatics analyses of *T*. *hominis* genome [8,15]. The mitosomal alternative respiratory pathway (light orange) provides the mechanism for oxidation of cytosolic NADH to NAD^+^; and the NAD^+^ serves as an electron acceptor required to maintain generation of ATP in glycolysis. **(E)** Model of the main mitosomal function at different stages of the *T*. *hominis* life cycle. The intracellular stage of the life cycle is initiated by the injection of the spore’s contents (sporoplasm) into the host cell, followed by the growth of the parasite cell into a large multinucleate proliferative cell (meront). The inset figures show the magnified section of the parasite cells containing a mitosome (orange). Mitosomes of the spores and the sporoplasms contain the machinery for oxidation of NADH to NAD^+^ via the alternative respiration (AOX and mtG3PDH), required for the generation of the ATP in the glycolytic pathway (glycolysis) [15]. Later stages of the *T*. *hominis* life cycle can utilize the membrane nucleotide transporters (NTTs) to steal ATP from their host [3–5], while using their mitosomes mainly for the generation of the Fe-S clusters [11,13] required by essential proteins such as DNA polymerases and helicases [13]. The Fe-S clusters are inserted into the apoproteins (APO) via the cytosolic Fe-S cluster assembly system (CIA) forming a functional holoenzyme (HOLO) [13]. Mitosomal proteins enriched in the spores are coloured in pink (AOX, mtG3PDH) whereas the protein detected across the parasite life cycle are coloured in brown (Fe-S biosynthesis including NFS), green (chaperone proteins e.g. mtHSP70), or blue (mitosomal protein import including TOM70). Fe-S containing nuclear proteins involved in DNA replication (DNA polymerases and helicases) were indicated in violet.

To determine the precise subcellular localization of AOX and mtG3PDH in *T*. *hominis*, we generated specific rabbit polyclonal antisera against these enzymes as well as rat antisera against the known mitosomal marker ThmtHSP70 [10]. To enrich for the specific antibodies, ThAOX- and ThmtG3PDH-sera were affinity purified against the respective recombinant proteins. In immunofluorescence microscopy experiments, the affinity purified ThAOX and ThmtG3PDH antibodies detected punctate signals that co-localized with those detected using ThmtHSP70 polyclonal sera (Figs 1A and S1), consistent with their localization to the mitosome. To further investigate their mitosomal localization, the ThAOX- and ThmtG3PDH-antibodies were used to probe western blots of the protein fractions obtained after separation by differential centrifugation (Figs 1B and S2). Similar to signals detected using antibodies against the known mitosomal marker proteins ThTOM70 [13] and ThmtHSP70 [10], ThAOX and ThmtG3PDH signals were detected mainly in the low-speed (1000 *g*) membrane fraction and in the mitosome-enriched [13] (25,000 *g*) fraction. In the mitochondria of model organisms, AOX localizes to the matrix face of the inner mitochondrial membrane [29], while mtG3PDH localizes on the intermembrane face of the inner membrane [30]. In order to investigate the location of ThAOX and ThmtG3PDH, the mitosome-enriched fraction was subjected to a proteinase-K protection assay (Figs 1B and S2). In agreement with previous reports [13], the mitosomal protein import receptor ThTOM70, which is located on the mitosome surface, was digested by proteinase (Fig 1B). By contrast, ThmtHSP70, ThAOX and ThmtG3PDH signals were protected from proteinase activity consistent with a protected location inside the mitosome.

### Mitosomal AOX and mtG3PDH are most abundant in *T*. *homnis* spores

To investigate the relative abundance of ThAOX and ThmtG3PDH in the dispersive extracellular *T*. *hominis* spores and in the intracellular stages of the parasite’s life cycle, we performed semi-quantitative western blotting analyses of protein extracts from purified spores and from a population of *T*. *hominis*-infected RK13 cells enriched in the intracellular stages (Figs 1C and S3), and immunofluorescence localization experiments in cryosection samples containing *T*. *hominis* spores (S15 Fig). The specific bands detected using antibodies against ThPGK (phosphoglycerate kinase), ThAOX, and ThmtG3PDH had significantly higher intensity in the extracts from spores than in the extracts from samples enriched in the intracellular stages of the parasite. This result supports a role for the mitosome in sustaining energy metabolism inside *T*. *hominis* spores. By contrast, signals for antibodies to components of the mitosomal ISC-biosynthesis pathway (ThNFS, ThmtHSP70) and the mitosomal protein import machinery (ThTOM70, ThmtHSP70) (Figs 1C and S3), were strongest in extracts from the intracellular stages of the parasite. These data are consistent with an increased requirement for iron-sulphur proteins in DNA replication and protein synthesis during rapid growth of the parasite, and with an increase in mitosome number prior to cell division into daughter cells, each requiring their own complement of ISC-producing mitosomes (Fig 1E).

**Fig 2:**
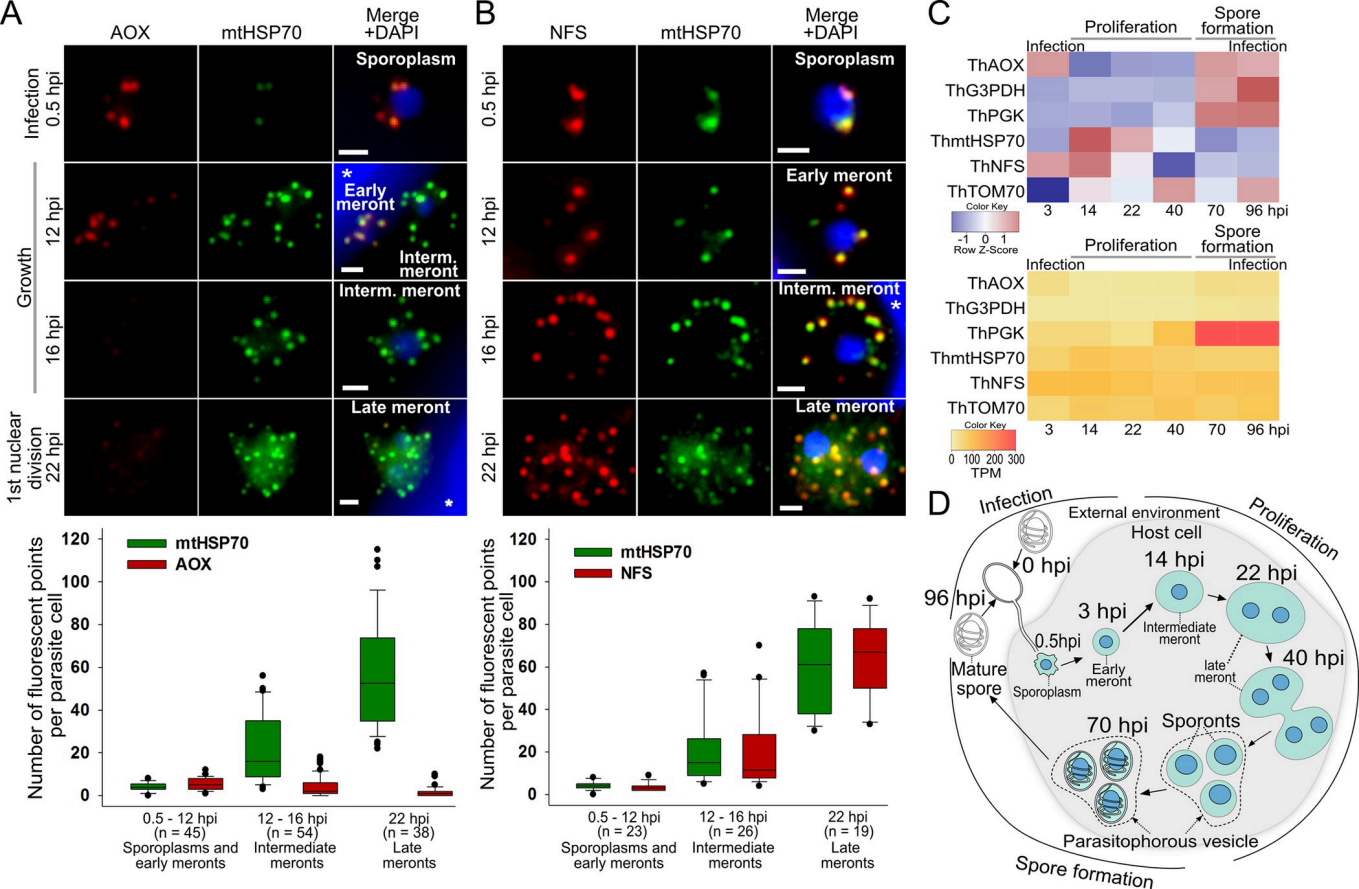
Detection of the AOX and mtG3PDH inside the *T*. *hominis* mitosomes at the early meront stages but not the late proliferative stages of the parasite’s infection cycle inside the RK13 host cells. **(A) and (B)** Representative immunofluorescence images of the *T*. *hominis* cells observed at the early time points of the time course of a synchronised *T*. *hominis* infection inside the RK13 cells. Methanol-acetone fixed samples were double labelled with the affinity purified antibodies against ThAOX (red, A top panel), or polyclonal sera against ThNFS (red, B top panel); and polyclonal rat sera against ThmtHSP70 (green; A, and B top panel). Presented time points correspond to: the first detection of the intracellular stages of the parasite 30 minutes after addition of the spores (0.5 hpi), increase of the parasite’s cell diameter (12 hpi, 16 hpi), and observation of double nucleate *T*. *hominis* cells following the first nuclear division (22 hpi). The observed stages of the parasite life cycle: sporoplasms, and meronts (early, intermediate, and late) were annotated on the images and described in detail in S4 and S5 Figs. The fluorescent images are maximum intensity projections of Z-stack across the parasite cells. Nuclei of the parasite and the host (asterisk) were labelled with DAPI, scale bars correspond to 1 μm. Analyses of the numbers of the fluorescent point signals detected with the specific antibodies against ThAOX (red, A bottom panel), ThNFS (red, B bottom panel); and ThmtHSP70 (green) inside the *T*. *hominis* cells at different stages of the parasite life cycle (A and B top panels, and S4 and S5 Figs). Extended version of the analyses including phase contrast images of the parasite cells, statistical analyses, and results obtained using antibodies against ThG3PDH are presented in S5 Fig. **(C)** Transcription levels of ThAOX, ThmtG3PDH, ThPGK, ThmtHSP70, ThNFS, and ThTOM70 from RNAseq analyses of samples from a synchronized time course of *T*. *hominis* infection sampled at the key intracellular stages of the parasite’s life cycle (D). The transcription levels were presented as TPM (transcripts per million) values (yellow-red scale), or as Z–score values (blue-red scale) to normalize for the differences between relatively highly transcribed PGK and the less abundant transcripts. **(D)** The model is based on previously published data [5] and presents the key stages of the life cycle of *T*. *hominis*: 0 hpi—the extracellular environmental spore (Mature spore) injects its contents (Sporoplasm) into the host cell using a specialised infection apparatus called a polar tube, 3 hpi–observation of small ovoid early meront cells, 14 hpi–increase in the parasite’s cell diameter and observed decreased number of the mitosomes labelled with anti-AOX/mtG3PDH antibodies, 22 h–first division of the parasite’s nuclei, 40 h–first division of the parasite’s cells, 70 h–initiation of the spore formation, 96 h–mature spores infect new uninfected host cells. During the Proliferation stage, the parasite is capable of stealing ATP from its host via nucleotide NTT transporters (inset figure). Dashed line indicates the membrane vesicle (parasitophorous vesicle) that surrounds parasite cells residing inside the host cytosol.

To test expression levels of the mitosomal proteins across different stages of the parasite’s life cycle inside the host, we used RNAseq analyses of RNA samples from a synchronised time course of *T*. *hominis* infection (Fig 2C). The transcriptomics analysis demonstrated that ThNFS and ThmtHSP70 were most highly transcribed during the proliferative stages of the parasite’s life cycle, consistent with the antibody data. ThPGK, ThAOX and ThmtG3PDH were most highly transcribed during formation of dispersive spores, further corroborating a role for these proteins in sustaining energy metabolism inside the spore (Fig 1D and 1E).

### Mitosomal respiratory enzymes are present inside mitosomes in early meront stages of *T*. *hominis* infection

In the RNAseq analyses, higher levels of AOX transcription were observed in the first sampled time point (Fig 2C), and AOX and mtG3PDH fluorescence was only detected in the smallest *T*. *hominis* early meront stage in the non-synchronised infection samples (S4E Fig). This suggested a possible role for the AR pathway in the initial stages of infection. To test this hypothesis, we investigated the mitosomal localization of AOX and mtG3PDH during the parasite life cycle in a synchronised time course of infection using immunofluorescence microscopy (Figs 2A–2B, S4 and S5). Antibodies against both proteins detected punctate signals co-localizing with mitosomal mtHSP70 in tiny (cell diameter of 2.25 μm ± 0.29 μm, S4B Fig) intracellular vegetative parasites shortly (0.5 hours post infection, hpi; Figs 2A–2B and S4A) after the addition of *T*. *hominis* spores to the uninfected host cell monolayer. These data suggest that AOX and mtG3PDH are already present in cells at the very early stages of infection, including both sporoplasm and early meronts through *de novo* expression and/or the prior presence of both proteins inside the spores. In previously published work, ThAOX and ThmtG3PDH were detected by proteomics of *T*. *hominis* spores, alongside glycolytic proteins including ThPGK, the enzyme catalysing the first ATP-yielding step of the pathway [15]. In the same study, ThPGK was also shown to be enriched in the spores of *T*. *hominis* using quantitative immunoelectron microscopy [15]. AOX and mtG3PDH were previously detected in the mitosomes of spores of *P*. *locustae*, using immunoelectron microscopy, but not in the intracellular stages [16]. Taken together the available data suggest that mitosomal ThAOX and ThmtG3PDH are present inside the infective spores and in the mitosomes of meronts, during the early hours of the infection process.

### Late-stage infection *T*. *hominis* mitosomes are enriched in ISC-biosynthesis proteins rather than respiratory enzymes

We next investigated if AOX and mtG3PDH could be detected inside mitosomes during the later stages of *T*. *hominis* infection. The number of fluorescent point signals of ThAOX and ThmtG3PDH decreased significantly in the larger parasite cells (Fig 2A bottom panel, and S5D–S5E Fig) after 12 hours post-infection (S4A Fig). By contrast, the number of mitosomal mtHSP70 signals observed in these larger cells was significantly higher than in cells from the earlier time points (Fig 2A bottom panel, and S5D–S5E Fig). The essential ISC biosynthesis protein Nfs was also localized to mitosomes across the life cycle of the parasite (Figs 2B and S5F), providing further support for the importance of mitosomes in this essential cellular process [11,13]. The absence of signals for AOX and mtG3PDH inside the later intracellular stages (12, 16, and 22 hpi; Figs 2A and S4A) could be caused by decreased protein expression levels combined with a dilution effect from division of mitosomes. This hypothesis is consistent with the decrease in the number of AOX transcripts (Fig 2C) after 14h post infection. The presence of AOX and mtG3PDH in the initial, but not the late stages of infection, may also explain the very low intensities of AOX and mtG3PDH, relative to the strong signals for ThmtHSP70 and ThTOM70, in western blots of the mitosome-enriched fraction (Figs 1B and S2). The samples used for fractionation were enriched in the intracellular stages of the parasite (based on microscopy observations), and the method used to lyse the cells (glass homogeniser) does not efficiently lyse spores. This suggests that the weak ThAOX and ThmtG3PDH bands in the 25k pellet may correspond to proteins present in the mitosomes of sporoplasm and early meront intracellular stages of *T*. *hominis*. These observations are consistent with a reduced role for the alternative respiratory pathway during the later stages of *T*. *hominis* cell proliferation.

In summary, the RNAseq and IFA evidence for ThAOX expression at early time points suggests that the enzyme can play a role in maintaining an optimal NAD^+^/NADH ratio to support glycolysis during the very early stages of infection when the parasite is establishing itself inside the host cell (Fig 1E). This may reflect a requirement of *T*. *hominis* to supplement ATP imported from the host by plasma membrane located nucleotide transport proteins [3–6] during host colonization. In addition to ATP generation, it is possible that early meronts of *T*. *hominis* may also use glycolysis and AOX to metabolise glyceraldehyde-3-phosphate from the pentose phosphate pathway, in order to generate NADPH required for thioredoxin, folate metabolism, and/or sphingolipid biosynthesis [8]. In contrast, late-stage infection *T*. *hominis* intermediate and late meronts seem to use their mitosomes mainly in the biosynthesis of essential iron-sulphur clusters rather than for alternative respiration (Fig 1E).

### Anti-microbial agents perturb the life cycle of *T*. *hominis* infecting mammalian cells

To investigate the possible role of mitosomal AR in *T*. *hominis* spores and early stages of infection we developed a methodology for testing the effects of specific inhibitors on parasites living inside the host. Functional studies of obligate intracellular parasites such as Microsporidia pose exceptional challenges because of the intimate association between the host and the parasite cells [32]. The use of specific inhibitors could potentially help in the functional characterization of microsporidian proteins that are important for infection, including enzymes mediating central roles of specific metabolic pathways. We used a recently developed methodology for establishing a synchronised time course of infection by *T*. *hominis* [5], to develop a protocol for testing the effects of specific drugs on parasites in conjunction with immunofluorescence microscopy and specific antibodies. We first tested the effects of two clinically used anti-microsporidian drugs [33]: namely albendazole [34,35] and fumagillin [21,36]. The concentrations of the drugs were guided by the published effects of fumagilin and albendazole on the microsporidian *Encephalitozoon intestinalis* in an *in vitro* RK13 host cell infection system [37]. In the previous study [37] the reported MIC50 values were: albendazole 8–55 ng/ml (30–207 nM), and fumagillin 0.5–0.8 ng/ml (1.1–1.9 nM), and the maximum inhibition of the infection was observed at albendazole 100 ng/ml (377 nM), and fumagillin 10 ng/ml (21 nM). In our experiments fumagillin was used at 9.2-higher concentration (200 nM, 92 ng/ml) to ensure observation of the maximum inhibition effects. The albendazole concentration used in our study was 92 ng/ml (350 nM). This was slightly below the previously reported concentration for full inhibition (100 ng/ml) of *E*. *intestinalis* as in the previous study higher concentrations of albendazole had deleterious effects on the host RK13 cells [37,38]. Albendazole was previously used against *T*. *hominis* in another study [38] where the concentration of 100 ng/ml (377 nM) appeared to be the minimum dose for control of *T*. *hominis* in mouse myoblast cell cultures. Similarly, we observed no secondary infections using 350 nM albendazole in our experimental system of *T*. *hominis* cultured in RK13 host cells. The concentration of the CCB (50 μM) was selected as the lowest concentration we tested where no progression of the *T*. *hominis* life cycle (parasite nuclear division, cellular division, or spore formation) was observed (S6C Fig).

Fumagillin is currently the only available broad spectrum anti-microsporidian drug, although it can also have toxic side effects [1]. The target of fumagillin inhibition, methionine aminopeptidase 2 (Met-AP2), catalyses the cleavage of N-terminal methionine [39], one of the most common post-translational protein modifications. Two classes of Met-AP enzymes (Met-AP1 and Met-AP2) are present in many eukaryotes, including humans and *Saccharomyces cerevisiae* [40]. In yeast, the deletion of both Met-AP homologues is lethal [41]. Fumagillin binds covalently to a histidine residue within the active site of human Met-AP2 [42] and inhibits growth of a Met-AP1 yeast deletion strain [36]. Although both Met-AP1 (Map1) and Met-AP2 (Map2) are present in the genome of *Rozella allomycis*, the cryptomycota outgroup to Microsporidia [9], only a homologue of the fumagillin-sensitive Map2 is present in microsporidian genomes. A crystal structure of the fumagillin-bound *E*. *cuniculi* Map2 is available [39] and 8 out of 10 residues contacting with fumagillin in *E*. *cuniculi* Map2, are also conserved in the *T*. *hominis* homologue (S7 Fig).

**Fig 3:**
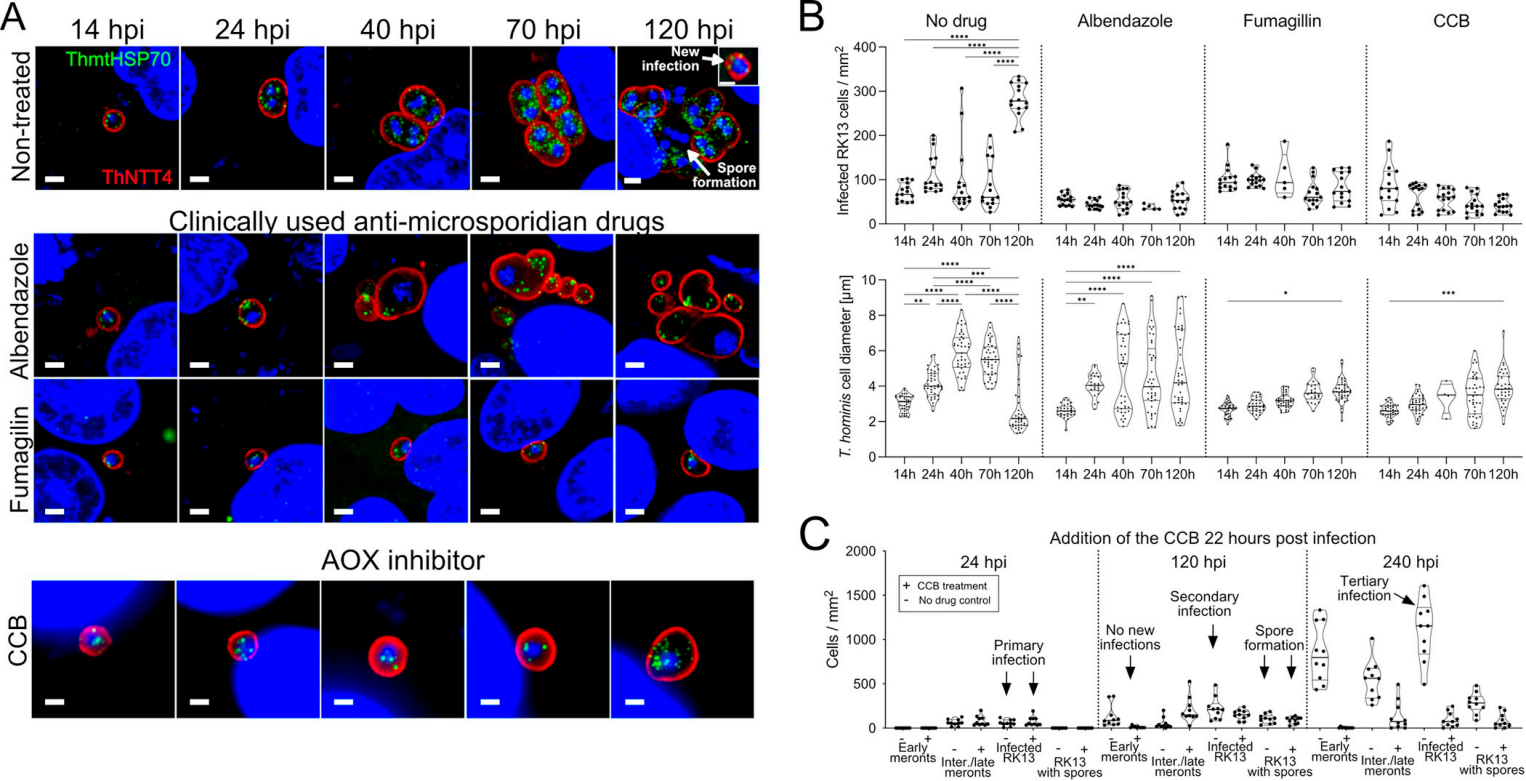
AOX inhibitor supresses growth of *T*. *hominis* cells inside the RK13 host cells at early meront stages of the infection, and affects the ability to infect by the spores formed in the presence of the drugs. Effects of the two clinically used anti-microsporidian drugs (albendazole, 350 nM; and fumagillin, 200 nM), and an inhibitor of the AOX Colletochlorin B (CCB, 50 μM) on progression of the parasite life cycle were tested in a synchronised time course of infection in *T*. *hominis-*RK13 infection system. The drugs were added to infective spores suspended in culture medium 24 hours prior to the initiation of infection. **(A)** Confocal immunofluorescence images of representative *T*. *hominis* cells labelled with rabbit antibodies against ThNTT4 (red) and rat antibodies against mtHSP70 (green); observed in samples collected at different time points of incubation with the drugs. The time points corresponded to: observed decreased number of the mitosomes labelled with anti-AOX/mtG3PDH antibodies (14h), first nuclear division (24h), first cellular division (40h), initiation of the spore formation (70h), infection of new host cells by newly formed mature spores (120h). New infections were first observed at 96h (Fig 2C); but dominated cultures and were quantified at 120 hours post infection. Nuclei of the parasite and the host were labelled with DAPI (blue), scale bars correspond to 2 μm (non-treated, albendazole, fumagillin) or 1 μm (CCB). **(B)** Violin plots of the numbers of the *T*. *hominis* infected RK cells (top row), or the diameter of *T*. *hominis* cells (bottom row) observed across a time course of infection in presence of the drugs (albendazole, fumagillin, and CCB), and in the non-treated control (No drug). Datapoints were displayed as black circles. ANOVA analysis was used to test significance of the differences between the measured values (****, P ≤ 0.0001; ***, P ≤ 0.001; **, P ≤ 0.01; *, P ≤ 0.05). The same no drug control was used for comparisons with the different treatment groups (S6 Fig). All experiments were performed in three biological replicates. **(C)** To test the effects of the CCB on the parasite development after the observed loss of the AOX and mtG3PDH mitosomal fluorescent signals (Figs 1D and 1F and S5) the drug was added 22 hours after the initiation of the infection (S8 Fig). Violin plots represent the numbers of the: *T*. *hominis-*infected RK13 cells (infected RK13), the RK13 cells containing bags of *T*. *hominis* spores (RK13 with spores), early *T*. *hominis* meronts (early meronts), and intermediate/late *T*. *homnis* meronts (inter./late meronts); observed across the time course of infection in the CCB-treated samples (+), or in the non-treated control (-).

To test the effects of fumagillin on *T*. *hominis*, we investigated the progression of the parasite life cycle inside RK13 host cells in fumagillin-treated and non-treated control cultures. In the non-treated control, *T*. *hominis* progressed through its life cycle, leading to the formation of mature spores 120 hpi (Fig 3A, non-treated). The spores that formed in the non-treated samples were able to infect new host cells, as evidenced by the observation of early meronts inside the newly infected host cells, and a significant increase in the numbers of infected host cells at 120 hpi. By contrast, only the very early stages of the *T*. *hominis* life cycle (early meronts) were observed (Fig 3A) in all time points from the samples treated with fumagillin. No parasites were observed undergoing nuclear division, cell division, or spore formation, suggesting arrest of the *T*. *hominis* life cycle at a very early stage of the infection. Consistent with the observed absence of newly formed spores, no new infections were observed at 120 hpi in the fumagillin-treated samples (Fig 3A). Despite the disruption of the parasite’s life cycle, drug treated *T*. *hominis* cells persisted inside the host cytosol and significantly increased their cell diameter, with the highest measured average change of 1 μm between the time points 14 hpi and 70 hpi (Fig 3B). In the corresponding time points of the non-treated control, the parasite’s cell diameter increased by on average 2.5 μm (the highest recorded change of 2.8 μm was measured between 14 hpi and 40 hpi) (Fig 3B). The measured increase in the parasite cell diameter in the fumagillin-treated samples indicated that *T*. *hominis* can still grow at a limited rate at this drug concentration. These data suggest that the inhibition was not complete—potentially due to the complex pharmacokinetics of the drug in the *T*. *hominis/*RK13 co-culture system. The observed inhibition of the parasite life cycle is consistent with an important role of *T*. *hominis* Map2 in post-translational protein modifications during the very early stages of infection, including early meronts.

The anti-microsporidian activity of albendazole is most likely due to its ability to bind to microsporidian beta-tubulin and inhibit the formation of microtubules [43]. In the albendazole treated samples, *T*. *hominis* cells increased their cell diameter and divided at 40 hpi, as in the non-treated control (Fig 3A). However, unlike the control, in drug-treated samples the cell division was asymmetric, leading to the formation of a single large ‘mother’ cell and single small ‘daughter’ cell (Fig 3A). DAPI-stained nuclei were observed in the ‘mother’ cells but not in the ‘daughter’ cells, indicating inhibition of the nuclear division process (Fig 3A). This is consistent with the proposed mechanism of albendazole activity, whereby binding of the drug to beta-tubulin inhibits formation of nuclear microtubules required for the division of the nucleus in the process of closed mitosis (where the nuclear envelope stays intact) [44] observed in Microsporidia [43]. The process of asymmetric division with the formation of anucleate ‘daughter’ cells continued throughout the time course of the infection (Fig 3A). We observed no evidence for newly formed spores inside the infected host cells, or any new infections, in the albendazole-treated 120 hpi samples. Although the albendazole treatment did not inhibit the ability of *T*. *hominis* to divide or increase in biomass, it inhibited nuclear division and the formation of new spores (Fig 3A and 3B) required for the propagation of the infection.

### AOX antagonists inhibit progress of the life cycle of *T*. *hominis* but do not inhibit AOX-negative *E*. *cuniculi*

We investigated if AOX antagaonist, colletochlorin B (CCB) [45] affected the life cycle of *T*. *hominis* infecting RK13 cells (Fig 3). CCB inhibited growth of the parasite in the early meront stages of the infection, reminiscent of the effects observed in the fumagillin-treated samples (Figs 3 and S9). In the treated samples, the parasite cell diameter increased significantly over the time course of infection; however, no nuclear division, cell division, spore formation, or new infections were observed; suggesting a microstatic rather than a microcidal effect of the drug.

To investigate if the observed inhibitory effect of CCB was reversible, the drug was removed by washing with culture medium without the drug 22h after the start of the infection (S10 Fig). After drug removal, the parasite continued to progress through its life cycle, suggesting that the microstatic effect is reversible, which was unexpected given the reported tight-binding of CCB to AOX *in vitro* [45]. This might be consistent with a continuous low-level expression of AOX in the early stages of infection as suggested by the RNAseq analyses (Fig 1D). The observed inhibition of the parasite’s life cycle in the presence of a specific AOX inhibitor suggests that mitosomal AOX plays an important role during the early meront stages of the infection.

In order to test if CCB has an effect on the progression of the parasite life cycle after the observed loss of mitosomal AOX fluorescent signals, CCB was added to the culture 22 hours after the addition of *T*. *hominis* spores to the host cells (Figs 3C and S8). Parasite cells in the CCB-treated samples grew, divided and made spores similar to the non-treated control. However, no new infections were observed in the presence of CCB, despite observing spore bags inside the host cells (S8 Fig). This suggests a functionally important role for AOX during spore formation and/or during the infection process. In the non-treated samples, the parasite life cycle was completed within 120 hpi and new infections were observed 120 and 240 hpi (Figs 3C and S9).

**Fig 4:**
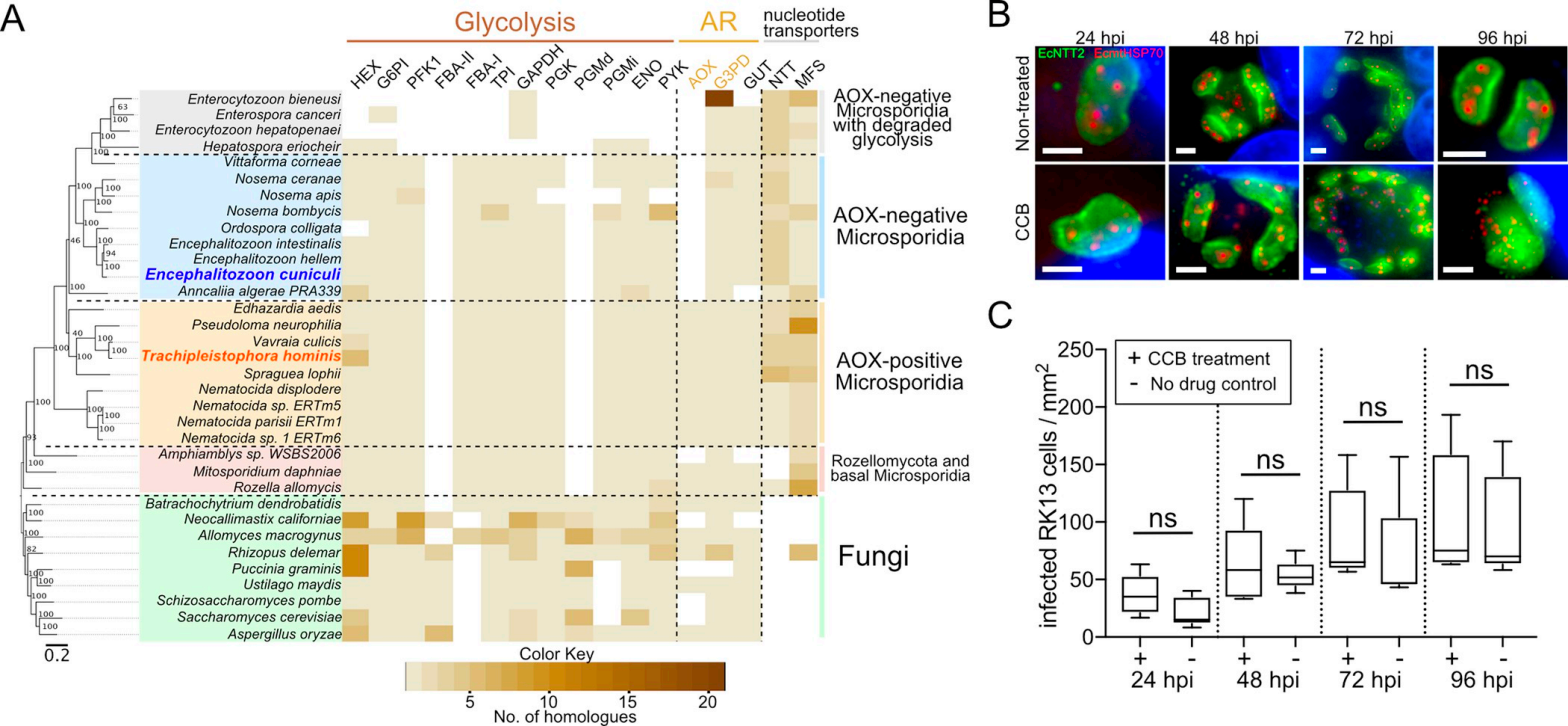
The AOX inhibitor CCB has no apparent effect on AOX-deficient microsporidian *E*. *cuniculi* infecting RK13 cells. **(A)** Heatmap representing distribution of enzymes involved in energy metabolism including glycolysis, alternative respiration (AR), and nucleotide transporters used to steal ATP from the host (NTT, and MFS) was mapped onto a species tree of Microsporidia, Rozellomycota, and Fungi. AOX-negative microsporidia including *E*. *cuniculi* were annotated in blue and grey. Presence or absence of protein homologues was verified in genomes using protein homology network analysis. The homologues of the two studied mitosomal AR proteins (AOX, G3PDH) are coloured in orange. The cytosolic glycerol-3-phosphate dehydrogenase (GUT) was indicated in black to distinguish it from the mitosomal AR enzymes. **(B)** Representative immunofluorescence images of the *E*. *cuniculi* cells, observed inside RK13 cells at different time points across the time course of synchronised infection in the presence of the Colletochlorin B derivative (CCB, 50 μM), or in the non-treated control (Non-treated). Sampled time point corresponded to: nuclear division (24h), cell division (48h), spore formation (72h), infection of new host cells by newly formed mature spores (96h). The methanol-acetone fixed samples were labelled with rat antibodies against *E*. *cuniculi* NTT2 [3] (green), and rabbit antibodies against *E*. *cuniculi* mtHSP70 [11] (red). Nuclei of the parasite and the host (asterisk) were labelled with DAPI (blue), scale bars correspond to 2 μm. **(C)** Box and whiskers plots of the numbers of the *E*. *cuniculi*-infected RK cells observed across a time course of infection in presence of the drug (CCB) or in the non-treated control (ND). T-test was used to test significance of the differences between the measured values (non-significant, ns).

The negative effects on the parasite cells were observed at relatively high concentrations of CCB (50 μM) which is much higher than the IC50 value of 14.8 nM for the AOX enzyme *in vitro* [45]. The *in vitro* activity of *T*. *hominis* ThAOX was also previously shown to be sensitive to 10 nM of the structurally related AOX inhibitor ascofuranone [18]. Under the *in cellulo* conditions of our assay it was not possible to measure the concentration of CCB inside the mitosome. To reach its target the drug has to cross at least four membranes (host cell membrane, parasite cell membrane, and two mitosomal membranes). The diffusion properties of CCB through these membranes and through the additional parasitophorous vesicle membrane, which surrounds *T*. *hominis* are unknown. That the cell cycle arrest we observe for *T*. *hominis* is due to the specific inhibition of ThAOX by CCB is supported by the absence of inhibition for the AOX-deficient microsporidian *Encephalitozoon cuniculi* (Figs 4A and S12) grown under the same conditions as *T*. *hominis* (Fig 4B and 4C).

## Conclusions

Our data demonstrate that key components of an AR pathway, AOX and mtG3PDH, are located in the *T*. *hominis* mitosome suggesting that in addition to the biosynthesis of the essential iron-sulfur clusters (ISC) [11,13], the minimal mitochondrion of *T*. *hominis* can regenerate NAD^+^ and by doing so contribute to maintaining an optimal NAD^+^/NADH ratio for effective cytosolic energy generation via glycolysis and possibly support other metabolic pathways. Consistent with previous data [18] the enzymes of the AR pathway appear to be present in *T*. *hominis* spores where they can support ATP-generation for house-keeping functions needed to preserve spore viability. Here we show that the AR pathway has a critical and previously unsuspected role in establishing the early meront stages of *T*. *hominis* infection of mammalian cells. The specific inhibition of the AR pathway stops the progression of the parasite life-cycle after infection, and prevents the formation of new spores needed to spread the infection to new host cells. The AR pathway appears to be less important once infection is established and surface-located nucleotide transporters (NTTs) can be used to steal host-generated ATP [3,5,6], and only reappears during the later stages of the parasite life cycle when spores are being formed. Notably three NTTs from *T*. *hominis* that are expressed on the cell surface during the proliferative stage can also import NAD^+^, in addition to ATP and GTP through a likely exchanger mechanism [5]. Hence these NTTs could contribute to the maintenance of optimal NAD^+^/NADH, ATP/ADP and GTP/GDP ratios required to drive *T*. *hominis* proliferative stage and the eventual differentiation into spores at the expense of the host cell. During this latter period the increased expression of AR pathway proteins is correlated with increased expression of glycolytic enzymes, ensuring that each spore contains a complete complement of proteins for ATP production in spores [15] and the early meront stages of infection. During the middle phase of active parasite growth and reproduction we did not detect signals for AOX or any drug-induced inhibitory effects on proliferating meronts, but we did detect the mitosomal cysteine-desulfurase Nfs, which is a core component of the pathway for mitochondrial ISC biosynthesis [11,13]. In *T*. *hominis* the ISC pathway appears to be important for the biosynthesis and export of an essential substrate for the biosynthesis of cytosolic Fe/S proteins, including enzymes that are essential for parasite growth and replication [13]. Despite the demonstrated importance of the AR pathway for *T*. *hominis*, some Microsporidia species that infect humans have lost AOX (e.g. *E*. *cuniculi*) or AOX and glycolysis *(Enterocytozoon bieneusi)* (S11 Fig). As we show here for *E*. *cuniculi* these species will not be susceptible to AR inhibitors. Like all canonical Microsporidia these two species also encode NTTs (Fig 4A) [5]. Two NTTs, likely exchangers, from *E*. *bieneusi* are able to transport NAD^+^ [5]. Hence, these transporters could contribute to maintaining optimal NAD^+^/NADH ratio throughout the proliferative stage of *E*. *bieneusi*. How indigenous energy generation is achieved and sustained in these two species in their spores is less clear. As champions of minimalism, some Microsporidia might have developed the capacity of storing sufficient energy to sustain the initial steps of the infection process.

In summary, our data suggest that the *T*. *hominis* mitosome has at least two critical functions in the parasite biology; to support the biosynthesis of essential cytosolic and nuclear proteins and to help sustain parasite indigenous energy production during key early meront and late spore-forming stages of the infection process. Our data demonstrate that the specific inhibition of this second function leads to the arrest of the infection progression, suggesting that it may be a valuable target for therapeutic intervention for Microsporidia containing the AR pathway.

## Materials and methods

### Protein expression and generation of antibodies

Full length *T*. *hominis* mtHSP70, and *T*. *homnis* mtG3PDH (orf_1639, GenBank: ELQ75445.1) gene excluding the first 393 nucleotides on the 5’ terminus and 21 nucleotides on the 3’ terminus, were cloned into a pET100 expression plasmid and expressed in the *E*. *coli* BL21 (DE3) as His-tagged recombinant proteins. The first 108 nucleotides excluded from the *Th*mtG3PDH construct, corresponded to the predicted N-terminal targeting signal which may be cleaved off during the import to the mitosomes [18], and thus may not be present in the mature mitosomal mtG3PDH. The remainder of the *Th*mtG3PDH sequence excluded from the construct, corresponded to the region of the *Th*mtG3PDH highly identical to the mammalian mtG3PDH, that could potentially cause cross-reactivity of the produced antibodies with the mammalian homologue in the rabbit kidney (RK13) host cells. A synthetic gene encoding *T*. *hominis* AOX sequence (orf_2158, GenBank: ELQ74912.1), excluding two regions (positions 421–510, and 601–690 of the nucleotide sequence; corresponding to the regions Tyr141-Leu170, and the Leu201-Phe230 in the amino acid sequence) homologous to the membrane interaction domains in the solved structure of *T*. *brucei* AOX [19] that may be poorly accessible to antibodies, was synthesized (GeneScript, USA), cloned into pQE40 expression plasmid, and expressed in the *E*. *coli* M15 as a DHFR fusion protein. Expressed proteins were purified with the BugBuster protein extraction reagent (Novagen), following the manufacturers protocol with the addition of Benzonase (Novagen) and lysozyme. 1 mg of each purified protein was separated by SDS-PAGE, and the Instant Blue stained protein bands were cut out from the polyacrylamide gels and used for the commercial (Agrisera, Sweden) generation of rabbit (*Th*AOX, and *Th*mtG3PDH), and rat (*Th*mtHSP70) polyclonal antisera. Each of the animals was immunized three times (week 1, week 5 and week 9) followed by the ELISA test and test bleeding (20 ml rabbit; 0.1 ml rat; week 11). The final immunization (week 13) was followed by the final bleed (50–70 ml rabbit; 3–4 ml rat; week 15).

To affinity-purify the antibodies, approximately 500 μg of protein used for immunisation of animals was separated on SDS polyacrylamide gels (100 μg/gel), transferred onto a nitrocellulose membrane, and blocked using mTBST (Tris-buffered saline; 0.1% Tween 20; 5% milk). 5 ml of the *Th*mtG3PDH, or *Th*AOX rabbit antisera were diluted 1:10 in mTBST and incubated with the membrane overnight at 4°C with rocking. After washing the membrane three times with TBST and once with TBS, the antibodies were eluted from the membrane with 0.2 M glycine-HCl, pH 2.5 for 10 minutes, followed by neutralisation to pH 7.0 using unbuffered 1M TRIS, and the addition of NaCl to final concentration of 50 mM. The purified antibodies were concentrated to the concentrations of 0.1–0.5 mg/ml (measured with NanoDrop, ThermoFisher) and the buffer was changed for PBS, using protein concentrator ultrafiltration centrifugal tubes (100,000 MWCO, ThermoFisher). The antibodies were aliquoted, stored frozen at -20°C, or in 50% glycerol at -20°C; and used at 5 μg/ml for IFA, or 1 μg/ml for Western blotting.

### Western blotting analyses of protein extracts from T. hominis spores and T. hominis infected RK cells

*T*. *hominis* infected- (ThRK13) and non-infected-Rabbit kidney cells (RK13) were routinely cultured in 175 cm^2^ flasks at 37°C, and 5% CO_2_ in Dulbecco’s Modified Eagle Medium (DMEM), containing 10% fetal bovine serum (Sigma), kanamycin 100 μg/ml, penicillin 100 μg/ml, streptomycin 100 μg/ml and fungizone 1 μg/ml. For total protein extraction, a confluent monolayer of ThRK13 or RK13 was washed three times with PBS and lysed with a 2% SDS in PBS lysis buffer containing protease inhibitor cocktail (Sigma P8340), PMSF (Sigma), MgCl_2_ (0.6 mM final concentration), and Benzonase (25U, Novagen). *T*. *hominis* spores were purified from the culture medium collected from ThRK13 culture, with density gradient centrifugation in Percoll as described previously [5]. The Percoll purified spores were lysed by boiling for 10 minutes in the 2% SDS in PBS lysis buffer with protease inhibitors. The concentration of the samples for western blotting was determined with Pierce BCA assay (ThermoFisher Scientific), and 10 μg of protein extracts in 1 x Laemmli sample buffer (1xSB) was loaded per lane for SDS–PAGE and transferred onto a nitrocellulose membrane for Western blotting analysis. All blocking and antibody incubations (primary overnight at 4°C, secondary HRP-conjugated 1h at RT) steps were performed in mTBST, and washes (3 x 10 minutes) were performed using TBST with the final wash in TBS. Proteins were visualized by chemiluminescence using a ChemiDoc XRS imager system (Bio-Rad). Protein transfer efficiency was verified by Coomassie Blue staining of the gels and Red Ponceau S staining of the membranes after blotting. Band intensities were analysed using Image Lab (Bio-Rad) and in FIJI [31].

### Immunofluorescence assay localization of microsporidian proteins and image analyses

Immuno-fluorescence assay (IFA) was done as described previously with minor modifications [5]. Shortly, RK13 cells infected with *T*. *hominis* or *E*. *cuniculi* grown on coverslips were fixed in methanol/acetone 50:50 v/v at -20°C for at least 10 minutes. After blocking with 5% (w/v) milk in PBS (mPBS), slides were incubated overnight with a mPBS solution containing the relevant affinity purified antibodies or antisera at 4°C, washed three times in PBS, and then incubated for 1 h with the secondary goat anti- rat or anti-rabbit antibodies (ThermoFisher) conjugated to Alexa Fluor 594 (red) or 488 (green). For the double labelling experiments, the incubation with the rat primary and the incubation with anti-rat secondary antibodies, were followed by the incubation with the rabbit primary and the incubation with the anti-rabbit secondary antibodies. Cells were incubated with DAPI in PBS for 5 min to visualize the host and the parasite nuclei. Slides were mounted using ProLong Gold (ThermoFisher). All cells were visualized using either a Nikon A1R confocal microscope with X63 or a Zeiss Axioimager II microscope with a X100 phase contrast objective lens. The images were processed in FIJI [31]. Fluorescent point signals were detected and quantified using “Find Spots” function implemented in Volocity (PerkinElmer). Colocalization of fluorescent point signals was tested using Coloc2 plugin in the FIJI [31] as described in S1 Fig.

### Testing effects of specific drugs across the time course of microsporidian infection in the RK13 cells

The fresh spores were isolated on the day of the experiments by lysing the 175 cm^2^ cultures of the ThRK13 or EcRK13 cells in 0.2% triton X-100 in PBS, followed by a sonication (3 x 45 seconds at low power intensity of 40 W, and 0.5 second cycling, on ice), and Percol purification of the highly resistant microsporidian spores. Gradient separation and removal of different stages of the spore formation, present in the lysates in addition to the infective mature spores, was avoided in order to minimise the loss of the infective spores. For *T*. *hominis* infection usually at least 1 flask of heavily infected RK culture was required for the infection of a single 24-well plate. In order to achieve the high levels of infection required for the isolation of high numbers of microsporidian spores, the RK13 cells already infected with the parasite had to be cultured for at least one month with the regular culture splitting (1:2–1:3 dilution at least once a week) and without the addition of the non-infected host cells, as the trypsinization of the ThRK13 or EcRK13 had a positive effect on the infection levels. To initiate the synchronised time course of the microsporidian infection, the Percol purified spores of *T*. *hominis* (5–20 x 10^6^ /cm^2^) or *E*. *cuniculi* (1–5 x 10^6^ /cm^2^) were added to the 80% confluent RK13 monolayer, and incubated for 2 hours at 37°C. The incubation of the spores was followed by a thorough washing (at least 3 washes) with the DMEM to remove the excess spores. Washing the cultures with the DMEM culture media instead of PBS was crucial to maximise the removal of the excess spores and minimise the stress to the host cells. For the immunofluorescence microscopy, theThRK13 or EcRK13 cells were cultured in 24-well plates on round cover slips, and time-points were collected by fixing the cells in methanol/acetone followed by the IFA. To test the effects of the drugs, the purified spores were added to a DMEM containing a drug (DMEMd) and incubated overnight at 4°C. After the overnight incubation, the spores in DMEMd were incubated for 1 hours at 37°C and added to a monolayer of the RK13 cells to initiate the infection (as described above). The DMEMd and DMEM media were changed every 48 hours. The drugs were dissolved in the DMSO (Sigma-Aldrich) and used at the following concentrations: albendazole (Alfa Aesar, 98% purity), 350 nM; fumagillin (Cayman Chemical; 95% purity), 200 nM; Colletochlorin B (CCB), 50μM. Non-treated controls were always grown in the presence of the DMSO concentration equivalent to that used in the treated samples. Every experiment was performed in triplicates and was independently replicated three times.

For the quantitative analyses of the infection, five, 4x4 mosaic images were taken using a Zeiss Axioimager II microscope with a X100 phase contrast objective lens at the top, the bottom, right side, left side, and in the centre of each cover slip. The parasite meronts were identified based on the labelling with rat anti-mtHSP70, rabbit anti-NTT4 antibodies, and DAPI. Due to the impermeability of the microsporidian spore to the antibodies, the intracellular bags of spores were identified based on the phase contras images. A single focus of the parasite infection (a single parasite cell, in early time points; or a group of parasite cells within a single parasitophorous vesicle, in later time points) was counted as a single infected host cell. The diameter of the *T*. *hominis* cells was measured at the widest area of the parasite cell labelled with the cell membrane marker (NTT4). Significance of the observed effects was tested using T-test; and ordinary two-way ANOVA (Tukey) in GraphPad Prism 9 (GraphStats), where tested drugs were the first factor and the time points of the infection were the second factor.

### Fractionation of infected rabbit kidney cells, and proteinase K treatment of the mitosome-enriched fraction

Fractionation and proteinase K protection assay were performed as described previously [5] with modifications. Shortly, *T*. *hominis* infected RK13 cells were washed in PBS, and scraped off the surface of the culture flask and suspended in an ice cold HSDP lysis buffer (0.25 M sucrose, 10 mM HEPES-KOH pH 7.2) containing a protease inhibitor cocktail (Sigma), PMSF (Sigma), DNase (1 U/ml), and RNase (10 mg/ml). All subsequent manipulations were performed on ice. Cells were lysed using a Dounce homogenizer, the lysate was incubated until not viscous, and subsequently subjected to ultracentrifugation at 1,000g for 10 min, 10,000g for 30 min, 25,000g and 100,000g for 1 h. The 100,000g supernatant represented the cytosolic fraction. The pellets were resuspended in PBS containing proteinase inhibitors and PMSF, with an exception of the 25,000g samples for the proteinase K protection assay, which were resuspended in PBS. The concentration of the samples for western blotting was determined with BCA assay, the samples were boiled in the 1xSB, and 10 μg of protein from each fraction was loaded per lane for SDS–PAGE and Western blot analysis using HRP-conjugated secondary antibodies (Jackson Laboratories). Proteins were visualized by chemiluminescence using a ChemiDoc XRS imager system (Biorad).

Proteinase K protection assay was performed on the 25,000g (mitosome-enriched) pellets. The 25,000g pellet was split into three aliquots which were: either incubated with 50 μg/ml of proteinase K (Roche) for 20 minutes, or incubated 0.2% (v/v) Triton X-100 for 10 min at room temperature followed by the incubation with the proteinase for 20 min; or incubated without the addition of the proteinase and the detergent (non-treated control). The proteinase treated samples as well as the non-treated control were then incubated with trichloroacetic acid at 20% (v/v) final concentration for 30 min on ice. Precipitated protein pellets were washed in ice cold acetone and solubilized in 1xSB containing proteinase inhibitors and PMSF. The protein fractions were subsequently analysed by western blotting.

### RNA sequencing of the samples collected across the synchronised time course of *T*. *hominis* infection

Fresh *T*. *hominis* spores were isolated from heavily infected RK cells on the day of the experiment, used to inoculate RK cells grown to 80% confluence in 150 cm^2^ round tissue culture dishes, washed at 2h post inoculation, and grown as previously described for testing the effects of drugs across the time-course. At 3, 14, 22, 40, 70 and 96 h post-inoculation, cells from two culture dishes were separately harvested in RNAprotect cell reagent and immediately frozen at -80°C. A third culture dish at each time point contained round cover slips, which were used for IFA to monitor the progression of infection. Total RNA was extracted from the cell pellet using a combination of TRIzol reagent and bead beating, as previously described [46]. Paired-end libraries were prepared using the Illumina mRNA sample preparation kit and sequenced on two lanes of an Illumina HISeq 2500. Transcript abundances were calculated with kallisto [47] and analysed using sleuth [48]. The sequence data were submitted to GenBank SRA database: RNA-Seq data: BioProject PRJNA278775 with the BioSample accession numbers SAMN11265032-SAMN11265043 (one accession for each of the two samples per time point post infection) [5,6].

### Bioinformatic identification of microsporidian protein homologues

Protein similarity networks were generated out of 184,600 protein sequences encoded in 9 fungi and 21 microsporidia/cryptomycota genomes (downloaded from the NCBI server); with BLAST [49], and quick edge file optimization implemented in EGN [50] using the following thresholds: BLAST (a = 12, e = 1e-05), simple link parameter (E-value threshold = 1e-05, hit identity threshold = 20%, minimum hit coverage = 20%, without the best reciprocal hit condition enforcement), and with the quick edge file creation (E-value threshold = 1e-05, hit identity threshold = 20%, minimum hit coverage = 20%, minimal hit length = 30 amino acids). The 12,022 identified networks were analysed using igraph [51] and dnet [52]⁠ packages in R [53], and visualized in Cytoscape [54]. Communities of the densely connected subgraphs within the largest protein network that contained 61,903 protein sequences (the second largest network contained 734 protein sequences), were isolated using the walktrap community detection algorithm implemented in the igraph package [51]. Sequences of the proteins involved in the metabolic pathways of interest in the analysed fungi were downloaded from KEGG. Networks containing proteins of interest were extracted and annotated using eggnog-Mapper v1 [55]. Heatmaps of the protein networks were generated using gplots package (https://CRAN.R-project.org/package=gplots) ⁠within R.

### Microsporidian species tree

To generate a species tree of the analysed Microsporidia and Fungi, single orthologues from 51 networks that contained only a single protein sequence from each species, were aligned using MAFFT [56], trimmed with trimAl [57], and concatenated with the Nexus module from the Biopython package [58] to a final alignment of 8076 amino-acid sites. Best fitting phylogenetic model was selected using ProtTest3 [59], and phylogenetic tree was generated under PROTGAMMALG model with 100 bootstrap replicates in RaxML [60]. Phylogenetic trees were visualised and annotated using FigTree [61].

## Supporting information

S1 Fig Investigating colocalization of mitosomal proteins detected using specific antibodies. Single confocal sections of samples labelled with rat polyclonal sera against ThmtHSP70 and affinity purified specific rabbit antibodies against ThAOX or ThmtG3PDH were acquired, processed, and analysed according to the manual of a Coloc2 plugin in Fiji [31]. Colocalization scatter plots and statistics (Pearson’s R value 1 indicates perfect correlation and 0 indicates no correlation) were generated using the Coloc2. Costes P-value of 1.00 indicates that the correlation measured between the analysed images was always higher than that between the analysed images and randomised controls.
(TIFF)

S2 Fig Western blotting analyses of the cell fractions from the differential centrifugation of *T*. *hominis*-infected RK13 cells lysates. Coomassie stained SDS-PAGE gel **(A)**; and full-size western blots probed with the anti-mtHSP70 (B), -mtG3PDH (C), and -AOX (D) antibodies; of the cell fractions from the differential centrifugation experiment presented in the Fig 1B. Molecular weights in kDa are annotated next to the protein ladder (L). Red arrowheads indicate parasite-specific bands, blue arrowheads indicate host specific bands (S3 Fig), and green arrowheads indicate bands corresponding to the proteinase K. The dark background observed in the anti-ThAOX and anti-ThmtG3PDH antibodies was due to the long exposure times (100 seconds and 60 seconds respectively) required to detect the bands, which is consistent with the low abundance of these mitosomal proteins in the intracellular stages, relative to that in the spore stages (Figs 1C and S3).
(TIFF)

S3 Fig Representative western blots used for the analyses of the band intensities. Coomassie stained SDS-PAGE gel of non-infected control RK13 cells (RK), *T*. *hominis* infected RK13 cells (RKTh) and spores from *T*. *hominis* (ThSpores) **(A)**; and western blots probed with antibodies against the mtHSP70 **(B)**, PGK **(C)**, NFS **(D)**, mtG3PDH **(E)**, TOM70 **(F)**, and AOX **(G)**; of the proteins extracts used for the analyses of the band intensities presented in the Fig 2D. Red arrowheads indicate parasite-specific bands, blue arrowhead indicates host specific band detected with anti-mtG3PDH antibodies. Black rectangles indicate areas of the western blots where the band intensities were measured. Molecular weights in kDa are annotated next to a protein ladder (L).
(TIFF)

S4 Fig Characterization of the different morphotypes of *T*. *hominis* cells during the initial proliferative stage of infection inside the RK13 host. Monolayers of *T*. *hominis* infected host cells sampled at different time points after the initiation of the infection (A, C, D), or from a mixed non-synchronised infection (E) were fixed with methanol-acetone and double labelled with the affinity purified polyclonal rabbit antibodies against *T*. *hominis* AOX (red, A, C), *T*. *hominis* mtG3PDH (red, E), or *T*. *hominis* NFS (red, D); and polyclonal rat antisera raised against *T*. *hominis* mtHSP70 (green, A-E)**. (A)** Immunofluorescence and phase contrast images of the *T*. *hominis* cells observed at different time points across the time course of the synchronised infection. **(B)** Three distinct morphotypes (top panel) of the parasite cells imaged across the time course of the infection (A) were identified based on: the number of the observed nuclei, and the proportion of the numbers of the quantified mitosomal AOX/mtG3PDH and mtHSP70 fluorescent points. Early meronts; observed mostly at 0.5–6 hpi, and infrequently at 12 hpi; had a single nucleus; cell diameter of 2.25 μm ± 0.29 μm; and all of their mitosomes were double labelled with AOX/mtG3PDH and mtHSP70 antibodies. Intermediate meronts; observed mainly between 12–16 hpi; had a single nucleus; cell diameter of 3.20 μm ± 0.39 μm; and most of their mitosomes were labelled only with mtHSP70/NFS antibodies. Late meronts; observed after 22 hpi; had usually 2 or 4 nuclei; cell diameter of 5.23 μm ± 0.95 μm; and virtually all their mitosomes were labelled only with mtHSP70/NFS antibodies. The fourth morphotype observed at early proliferative stages of infection was a sporoplasm (A and S5 Fig, 0.5 hpi), characterised by a single nucleus; cell diameter generally smaller than that of an early meront; and amorphous shape as opposed to the ovoid shape of the meronts. The sporoplasms most likely corresponded to the contents of the spore injected into the host cell that initiates the infection. Sporoplasms were notoriously difficult to image due to their small size, extremely weak DAPI signal (relative to that of the host nuclei), and due to the presence of residual parasite debris stuck to the surface of the host cells (despite thorough washing) observed for the first few hours after the initiation of infection by the addition of the spores. Diameter of the parasite cells was plotted against the numbers of mtHSP70 point signals (bottom panel) measured for the cell morphotypes observed in the different time points of the time course of infection. Cell diameter was always measured across the widest part of a cell. **(C, D)** Images of all three characterized morphotypes labelled with anti- mtG3PDH (C) or anti-*T*. *hominis* NFS antibodies (D). **(E)** An uncommon image of all three morphotypes observed next to each other inside a single host cell in a sample from a mixed non-synchronised infection. All fluorescent images are maximum intensity projections of the Z-stack across the parasite cells. Scale bars correspond to 1 μm.
(TIFF)

S5 Fig Quantification of fluorescent points in mixed population of *T*. *hominis* infecting RK cells. Comparison of the numbers of fluorescent signals detected in different samples, such as different time points from a time course of infection experiments, can be affected by a sample-to-sample variation due to factor such as differences in thickness of the host cell monolayer, or small variations in the sample preparation (e.g. fixation temperature) despite following the same protocol. It can be particularly challenging when trying to compare numbers of signals that display a life-cycle stage specific labelling pattern, such as those detected using anti-AOX and anti-mtG3PDH antibodies. In order to directly compare different stages of the parasite life cycle in a single sample, numbers of fluorescent point signals were quantified (D-F) in randomly sampled fluorescent images of non-synchronised mixed population of *T*. *hominis* (S13 Fig). All morphotypes identified in the time course of infection (A-C) were also found in the samples of mixed population (S4E and S13 Figs). Only images that contained at least two out of three major morphotypes (early meronts, intermediate meronts, and late meronts) in a single field of view were selected for the analyses. Average number of the red (AOX) and the green (mtHSP70) fluorescent point signals was measured for a total of 137 cells observed in 20 z-stacks from two independent biological replicates corresponding to two independently infected and cultured populations of *T*. *hominis* infected RK13 cells (10 z-stack for each of the replicates). No statistically significant differences were found between the replicates for any of the measurements (T-test). Numbers of the analysed parasite cells (n) were displayed below the plots. The significance (T-test) of the differences in number of the detected fluorescent points was displayed above the plots (*** P ≤ 0.001, ** P ≤ 0.01, * P ≤ 0.05, not significant P > 0.05).
(TIFF)

S6 Fig Investigating the effects of fumagillin, albendazole, and CCB treatment on *T*. *hominis* cells grown inside the RK13 host cells. **(A, B)** Violin plots of the numbers of the *T*. *hominis* infected RK cells (A), or the diameter of *T*. *hominis* cells (B) observed across a time course of infection in presence of the drugs (albendazole, fumagillin, and CCB), and in the non-treated control (No drug). The displayed data is the same as that in Fig 3 but was presented relative to the non-treated control. Datapoints were displayed as black circles. ANOVA analysis was used to test significance of the differences between the measured values (****, P ≤ 0.0001; ***, P ≤ 0.001; **, P ≤ 0.01; *, P ≤ 0.05). All experiments were performed in three biological replicates. **(C)** Confocal immunofluorescence images of representative *T*. *hominis* cells labelled with rabbit antibodies against ThNTT4 (red) and rat antibodies against mtHSP70 (green); observed in samples collected at 2h, 24h, and 48h time points post infection in samples treated with CCB at concentrations required for either partial (10 μM) or complete (50 μM) inhibition of the parasite life cycle, relative to the non-treated control (no drug).
(TIFF)

S7 Fig Multiple sequence alignment of Map1 and Map2 homologues. Sequences of Map1 and Map2 from *S*. *cerevisiae*, *H*. *sapiens*, and *R*. *allomycis;* and Map2 from microsporidia were aligned using Muscle [62].The residues contacting the fumagillin and TNP470 in the crystal structure of *E*. *cuniculi* Map2 (PDB 3FM3) [39] were indicated (red arrowheads).
(TIFF)

S8 Fig Testing the effects of late addition of the coletochlorin B to the culture of RK13 cells infected with *T*. *hominis*. **(A)** Images of representative *T*. *hominis* cells observed in the samples collected across the time course of infection where, colletochlorin B (CCB) was added to the medium 22 hours after the addition of spores to the non-infected host cells, when AOX and mtG3PDH labelled mitosomes were absent from virtually all parasite cells (S5 Fig). Representative cells of the parasite life cycle stages observed at each time point; meronts (54 hpi), sporonts (64–80 hpi), sporoblasts (64–120 hpi), and spores (80–130 hpi); were indicated on the images (white arrows). Time points displayed above the images refer to the time points after the addition of the spores. **(B)** Low magnification image of the CCB treated samples at 136 hpi. All infected host cells contained spore bags (white arrows), but no new infections were observed. Cells labelled with antibodies represent late meront stages from the initial infection that have not entered the spore formation stage of life cycle. All samples were double-labelled with the antibodies against ThNTT4 (red), and ThmtHSP70 (green).
(TIFF)

S9 Fig Representative images of the *T*. *hominis-*infected RK13 monolayer, with and without the addition of CCB, 120 hours post infection. 120 hours after the addition of spores, in the culture of the *T*. *hominis* infected RK13 host cells treated with the CCB (top panel), only the persistent small *T*. *hominis* meronts (white arrowheads) from the initial infection were observed. At the same time point in the non-treated control (bottom panel), significantly higher number of the small meronts and sporoplasms (white arrowheads) from the secondary infection were observed. Smaller number of the heavily infected host cells from the primary infection (dashed circles); filled with the infective spores, and other stages of the parasite life cycle; were observed alongside the newly infected host cells. The heavily infected RK13 cells were characteristically multi-nucleated, with the host nuclei surrounding the foci of the infection. High intensity of fluorescence observed in the heavily infected cells is due to longer exposure times required to image the sporoplasms labelled with NTT4. The difference in the fluorescence intensity between the sporoplasms and late meronts observed in the same image is likely a result of an accumulation of the NTT4 across the intracellular stage of *T*. *hominis* life cycle. NTT4 belongs to the top 10% most abundant transcripts measured across the parasite life cycle [5], and the apparent high levels of NTT4 in the cell membrane of the larger late stage meronts are consistent with its proposed critical role in supporting the rapid parasite growth [5]. All samples were double-labelled with the antibodies against ThNTT4 (red), and ThmtHSP70 (green). Signal in red channel was highly exposed in order to visualize the small meronts and sporoplasms labelled weakly relative to the high intensity of labelling observed for the intermediate and late meronts.
(TIFF)

S10 Fig Testing the effects of the removal of the coletochlorin B from the culture of RK13 cells infected with *T*. *hominis*. Colletochlorin B (CCB) was added to the culture medium containing *T*. *hominis* spores and incubated at 4°C for 16 hours, followed by at least 1 hour of incubation at 37°C prior to the addition of the medium containing spores and the drug to the monolayer of uninfected RK13 cells. 22 hours post infection, medium containing the CCB was replaced with a culture medium without the drug. **(A)** Images of representative *T*. *hominis* cells observed in the samples collected across the time course of infection where, the CCB was removed at 22 hpi. Representative cells of the parasite life cycle stages observed at each time point; meronts (54–136 hpi), sporonts (64–136 hpi), sporoblasts (80–136 hpi), spores (120–136 hpi), and sporoplasms (136 hpi); are indicated on the images (white arrows). Time points displayed above the images refer to the time points after the addition of the spores. **(B)** Low magnification image of the CCB treated samples at 136 hpi. Heavily infected host cells containing meronts, spore forming stages (sporonts and sporoblasts), and spores are indicated with the dashed circles. White arrows indicate sporoplasms inside newly infected host cells (Merge), or spore bags inside the host cells infected during the primary infection (Merge + PC). All samples were double-labelled with the antibodies against ThNTT4 (red), and ThmtHSP70 (green).
(TIFF)

S11 Fig Evolutionary history of microsporidian adaptations to the intracellular parasitic lifestyle and potential impacts on drug resistance and sensitivity. Inferred gene gain (Fig 2A) of the ancestral nucleotide NTT transporter (turquoise), as well as inferred gene losses (Fig 2A) of key pathways for energy generations (magenta) were indicated on the cladogram of microsporidia. In the table, coloured squares indicated presence (blue) or absence (red) of genes and pathways for energy generation in genomes of microsporidia and *R*. *allomyces*. Presence or absence of certain genes, or amino acid residues in protein sequences, can be associated with sensitivity to antimicrobial agents in microsporidia. Sensitivity to the AOX inhibitor CCB is associated with presence of the AOX gene (this study). Sensitivity to the fumagillin is likely to be due to the absence of the Map1 gene [39] from the microsporidian genomes (S7 Fig), and the sensitivity to the albendazole is associated with a presence of a specific residues in Beta-tubulin amino acid sequence [63,64] (S16 Fig).
(TIFF)

S12 Fig Characterization of the *E*. *cuniculi* cells observed inside the RK13 host cells during the time course of infection. **(A, C)** As opposed to the *T*. *hominis* infection, where no clear organisation of the infection foci can be observed, *E*. *culiculi* develops inside the parasitophorous vesicle (turquoise arrows) throughout its life cycle, with a clear stratification of the different life cycle stages across the vesicle. Meronts (white arrowheads) are always observed at the edge of the vesicle; and are strongly labelled with the anti-EcNTT2 antibodies (green), and anti-EcmtHSP70 antibodies (red). Deeper inside the vesicles sporonts (magenta arrowheads) are clearly visible in the phase contrast images, and their mitosomes are labelled with the anti-EcmtHSP70 antibodies, but no labelling with the anti-*Ec*NTT2 antibodies can be observed. The sporoblasts and spores (red arrowheads) are not labelled with either EcmtHSP70 or EcNTT2 antibodies. The fluorescence images in the panels **A** and **C** are the same as in the Fig 4. **(B**, **D)** Low magnification images of the CCB-treated (B) or non-treated (D) *E*. *cuniculi*-infected RK13 culture at 96 hpi. *E*. *cuniculi* meronts, spore forming stages, and newly formed spores were observed inside the host cells infected during the primary infection (dashed circles). The host cells infected during the secondary infection by the newly formed spores contained only the meronts (white arrowheads). The white arrows indicated *E*. *cuniculi* nuclei which were often more clearly visible in the phase contrast images than in the DAPI-labelled (blue) fluorescence images, due to the weak intensity labelling of small *E*. *cuniculi* nuclei relative to the high intensity DAPI-labelling of the host nuclei (asterisk).
(TIFF)

S13 Fig Quantification of the mitosomes labelled with specific antibodies. **(A)** Z-stack of a representative field of view used for the quantification of the fluorescent points (Figs 1F and S5) detected with the affinity purified antibodies against ThAOX (red) and polyclonal sera against ThmtHSP70 (green). The images in the red and the green channel were highly overexposed in order to visualize the lowest intensity points. The exposure times and the thresholds were set for all samples so that no signals were detected in the controls prepared the same way as the analysed samples but without the primary antibodies, and so that the highest intensity points were not overlapping. Dashed lines indicate the areas of single cells identified based on the phase contrast image. **(B)** Representative images of the *T*. *hominis* mitosomes labelled with the ThmtHSP70 (green) antibodies, sampled across the parasite cell in the Z-axis at the optimum sampling distance automatically set using the Apotome.2 ZEN software (Zeiss). Blue Xs indicate position of the fluorescent points detected using Volocity’s (Perkin Elmer) ‘local intensity maxima’ detection algorithm (‘Find spots’ function). Thresholds were adjusted so that only points which were not overlapping, were detected in all cells within a single field of view. Widefield fluorescence microscopy (WFM) was selected over laser confocal scanning microscopy (LCSM) based on a number of factors, mainly: no observed photobleaching; good signal to noise ratio; and the ability to image all parasite cells in phase contrast, which was crucial for the rigorous classification of the different morphotypes of the parasite cells. The photobleaching using the LCSM was especially high in many parasite cells labelled with the anti-ThAOX and the anti-ThmtG3PDH antibodies, likely due to the low abundance of these mitosomal proteins, which is consistent with the low intensity of the western-blot bands detected using the same antibodies against the protein extracts from the samples enriched in intracellular stages of the parasite (Figs 1B, S2C, S2D,S2F, S3E and S3G). The main disadvantage of the WFM, especially relative to the super-resolution LCSM, is the lower resolution leading to the possible underestimation of the number of the mitosomes especially in the larger late and intermediate meronts (S14 Fig). This limitation had no significant effect on this study, mainly due to the clear decrease in the numbers and apparent intensities of the fluorescent points detected in the late and intermediate meronts using the anti-ThAOX and the anti-ThmtG3PDH antibodies (S4 and S5 Figs).
(TIFF)

S14 Fig Visualization of the mitosomes using super-resolution confocal microscopy. **(A)** Single confocal section of two adjacent late *T*. *hominis* meronts imaged using regular laser confocal scanning microscopy (LCSM, right panel), and super resolution STED (left panel) modes of Leica SP8 STED microscope. **(B)** Overlay of the STED (red), and the regular LCSM (green) images from (A). **(C, D)** Measurements of the diameter of a single florescent point (white line), or of the distance between the centres of two fluorescent points (turquoise line), measured in the STED image (D). The higher resolution of the STED often resolves a single fluorescent point observed in the confocal into two adjacent points. The STED microscopy also allows imaging of the mitosomes at the resolution closer to their size measured in electron microscopy images (∼50 nm x 90 nm) [10]. Despite at least two times lower resolution of the diffraction limited microscopy (regular LSCM, and wiedefield microscopy) the results obtained using these methods provided a good approximation for the analyses of variation in the numbers of the mitosomes in the different stages of the parasite life cycle observed in this study.
(TIFF)

S15 Fig Immunofluorescence images of cryo-sections of the environmental and intracellular *T*. *hominis* spores labelled using specific antibodies. Due to the difficulty in penetrating the microsporidian spore wall with the antibodies in samples fixed using either Methanol-Acetone or PFA-detergent fixation-permeabilization methods, in effort to label the interior of the spores we decided to use thin cryo-sections of the spores purified from the medium collected from the culture of *T*. *hominis* infected RK13 cells monolayer (A and B), as well as the cryo-sections of the *T*. *hominis* infected RK13 monolayer (C and D). The spores were purified using Percol gradient, and the spore pellets were suspended in the O.C.T. (optimal cutting temperature) compound and frozen in liquid nitrogen. The monolayers were, washed with PBS, scraped off the culture flask surface using a cell scraper, and suspended in the OCT. 3 μm cryo-sections of the samples were cut using cryo-microtome (Leica), placed on microscopy slides, and processed according to the standard immunofluorescence protocol (Material and Methods). **(A)** Environmental (i.e. collected from the medium covering the infected host cells) spores labelled with the affinity purified antibodies against ThAOX (red), and ThmtHSP70 (green), and imaged in phase contrast (PC); were found in the sectioned samples of the purified spores. **(B)** The majority of the spores were not labelled with the antibodies suggesting a low efficiency of sectioning. Furthermore, debris were observed throughout the sample. The debris were often labelled with the specific antibodies suggesting they may be cellular debris from the germinated and/or damaged spores. The spore apparently labelled with the specific antibodies (A) was annotated with the white arrowhead. **(C)** Intracellular spores labelled with the affinity purified antibodies against Th mtG3PDH (red), and ThmtHSP70 (green) and imaged using the differential interference contras (DIC) observed inside the host cell (white arrowheads). **(D)** Heavily infected host cell filled with the *T*. *hominis* spores was outlined with the dashed line. A single meront (magenta arrow) labelled with the anti-ThmtHSP70 antibodies, and two intracellular spores labelled with the anti-ThmtHSP70, and the anti-ThmtG3PDH antibodies (white arrows, and C) were observed inside the RK13 host cell. As in the case of the samples of purified spores, only a small fraction of the intracellular spores was labelled with the specific antibodies. Consistent with the hypothesis that this is due to the low cutting efficiency, the labelled spores were only observed at the top and the bottom (Z-axis) of the cell sections, Z-positions corresponding to the surfaces cut with the microtome knife. According to the microscopy measurements the distance between the top and the bottom of the samples was approximately 10 μm, which is considerably more than the setting of 3 μm used on the cry-microtome. These differences in thickness could explain the low efficiency of spore labelling, as only a fraction of spores at the top and the bottom of the sectioned material were sectioned. DAPI-stained (blue) RK13 host cell nuclei were indicated with asterisks. In the images of the sectioned spores (A and C) subpopulations of the fluorescent points double-labelled with both antibodies (yellow), as well as subpopulations single-labelled mainly with the ThAOX/ThmtG3PDH (red) were observed. Although, it is difficult to determine if the single labelled points represent a real population of mitosomes in which ThmtHSP70 was absent, or present at concentrations below the detection limit; or if they were observed due to the contamination from the surrounding material, or due to the damage to the spore’s ultrastructure during the sectioning of the samples. Presence of the subpopulation of the mitosomes enriched in the components of the alternative respiratory pathway cannot be excluded based on this data, and is supported based on the significantly higher number of the anti-ThAOX and anti-ThmtG3PDH labelled mitosomes that that of the anti-mtHSP70 labelled mitosomes, detected in the *T*. *hominis* sporoplasms and early meronts (S5E and S5D Fig). The image of the sporoplasms and of the early meronts containing examples of single-labelled mitosomes can be observe in the 0.5 hpi time point (S5A and S5B Fig).
(TIFF)

S16 Fig Multiple sequence alignment of microsporidian beta-tubulin. Amino acid sequences of microsporidian Beta-tubulin were aligned using Muscle [62]. The residues reported to be associated with the benzimidazole sensitivity [63,64] were indicated (red arrowheads).
(TIFF)
