# Supplementary figures and images for: Inhibition of mitosomal alternative oxidase causes lifecycle arrest of early-stage Trachipleistophora hominis meronts during intracellular infection of mammalian cells
Source: PLoS Pathog. 2022 Dec 20;18(12):e1011024. doi: 10.1371/journal.ppat.1011024 (PMC9767352; doi:10.1371/journal.ppat.1011024)

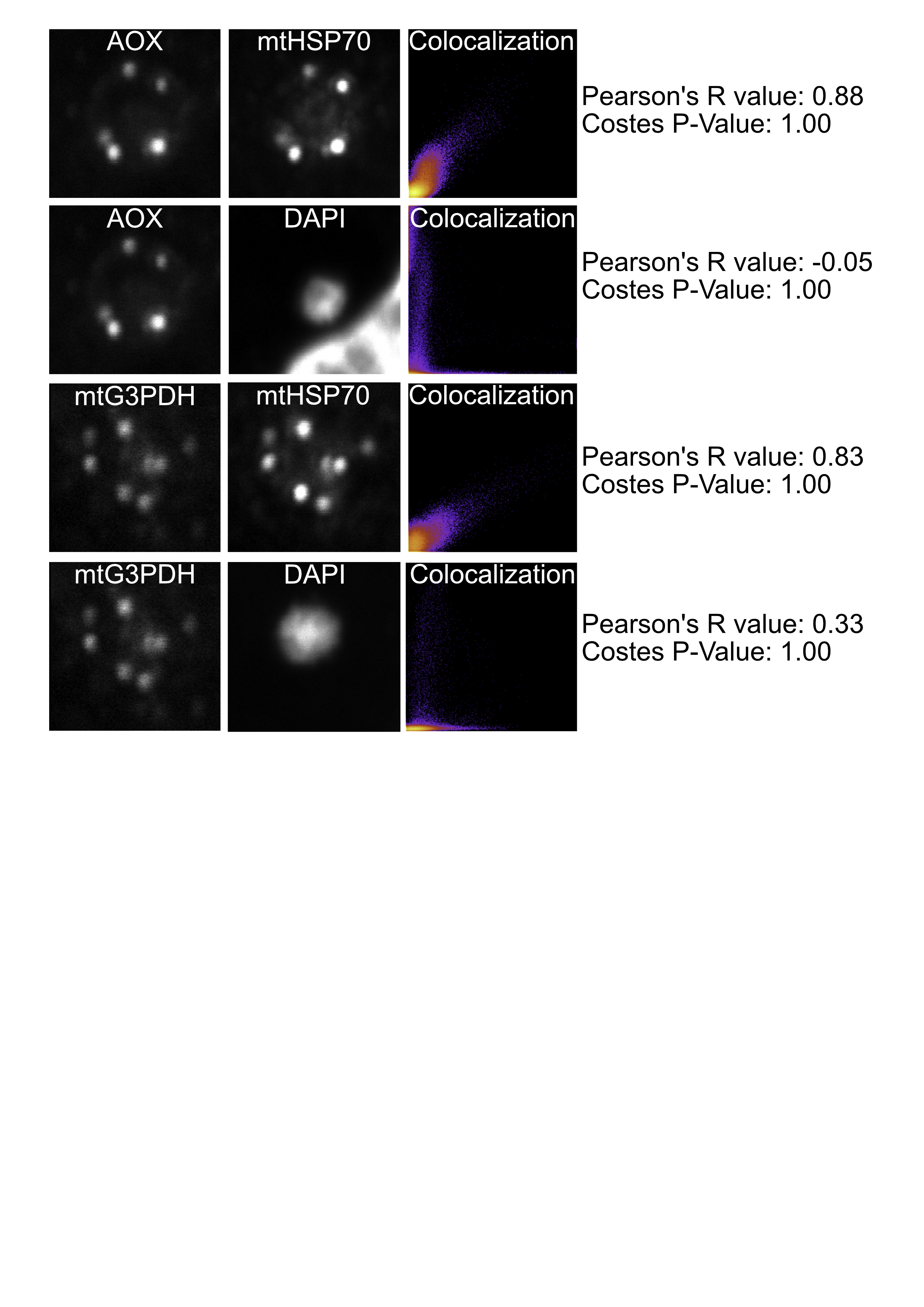

Supplement: S1 Fig — Single confocal sections of samples labelled with rat polyclonal sera against ThmtHSP70 and affinity purified specific rabbit antibodies against ThAOX or ThmtG3PDH were acquired, processed, and analysed according to the manual of a Coloc2 plugin in Fiji [31]. Colocalization scatter plots and statistics (Pearson’s R value 1 indicates perfect correlation and 0 indicates no correlation) were generated using the Coloc2. Costes P-value of 1.00 indicates that the correlation measured between the analysed images was always higher than that between the analysed images and randomised controls. (TIFF) [file ppat.1011024.s001.tiff]

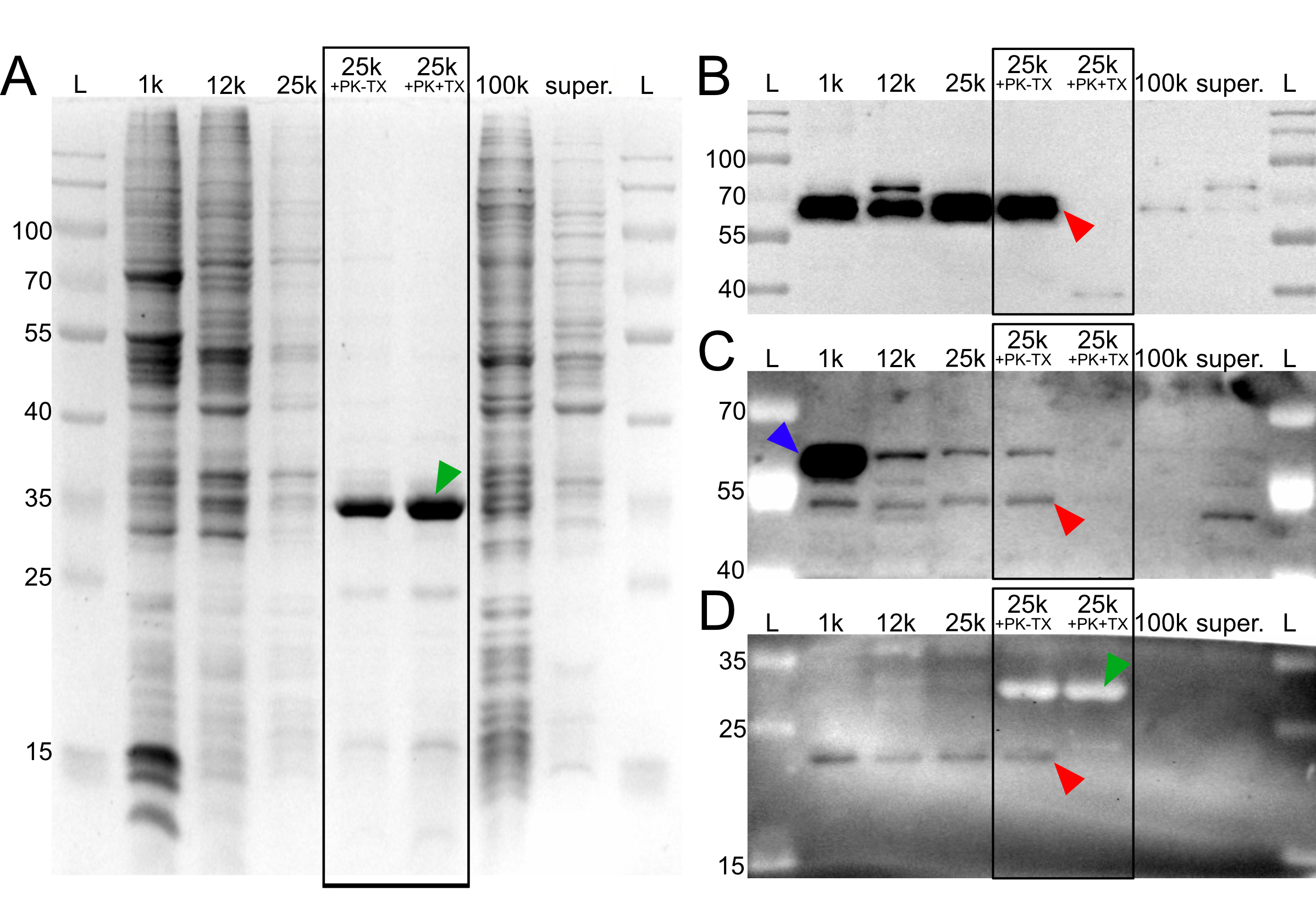

Supplement: S2 Fig — Coomassie stained SDS-PAGE gel (A); and full-size western blots probed with the anti-mtHSP70 (B), -mtG3PDH (C), and -AOX (D) antibodies; of the cell fractions from the differential centrifugation experiment presented in the Fig 1B. Molecular weights in kDa are annotated next to the protein ladder (L). Red arrowheads indicate parasite-specific bands, blue arrowheads indicate host specific bands (S3 Fig), and green arrowheads indicate bands corresponding to the proteinase K. The dark background observed in the anti-ThAOX and anti-ThmtG3PDH antibodies was due to the long exposure times (100 seconds and 60 seconds respectively) required to detect the bands, which is consistent with the low abundance of these mitosomal proteins in the intracellular stages, relative to that in the spore stages (Figs 1C and S3). (TIFF) [file ppat.1011024.s002.tiff]

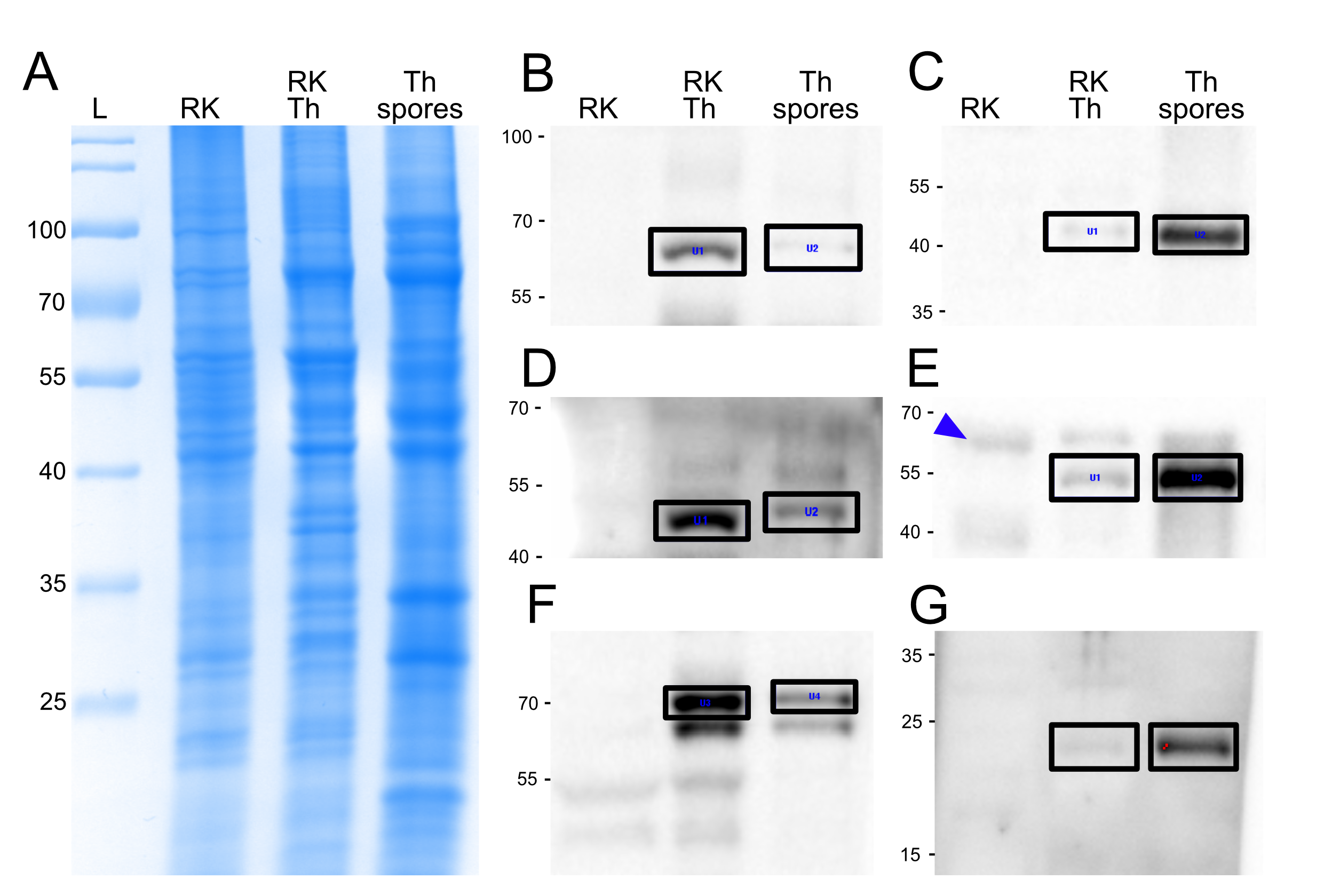

Supplement: S3 Fig — Coomassie stained SDS-PAGE gel of non-infected control RK13 cells (RK), T. hominis infected RK13 cells (RKTh) and spores from T. hominis (ThSpores) (A); and western blots probed with antibodies against the mtHSP70 (B), PGK (C), NFS (D), mtG3PDH (E), TOM70 (F), and AOX (G); of the proteins extracts used for the analyses of the band intensities presented in the Fig 2D. Red arrowheads indicate parasite-specific bands, blue arrowhead indicates host specific band detected with anti-mtG3PDH antibodies. Black rectangles indicate areas of the western blots where the band intensities were measured. Molecular weights in kDa are annotated next to a protein ladder (L). (TIFF) [file ppat.1011024.s003.tiff]

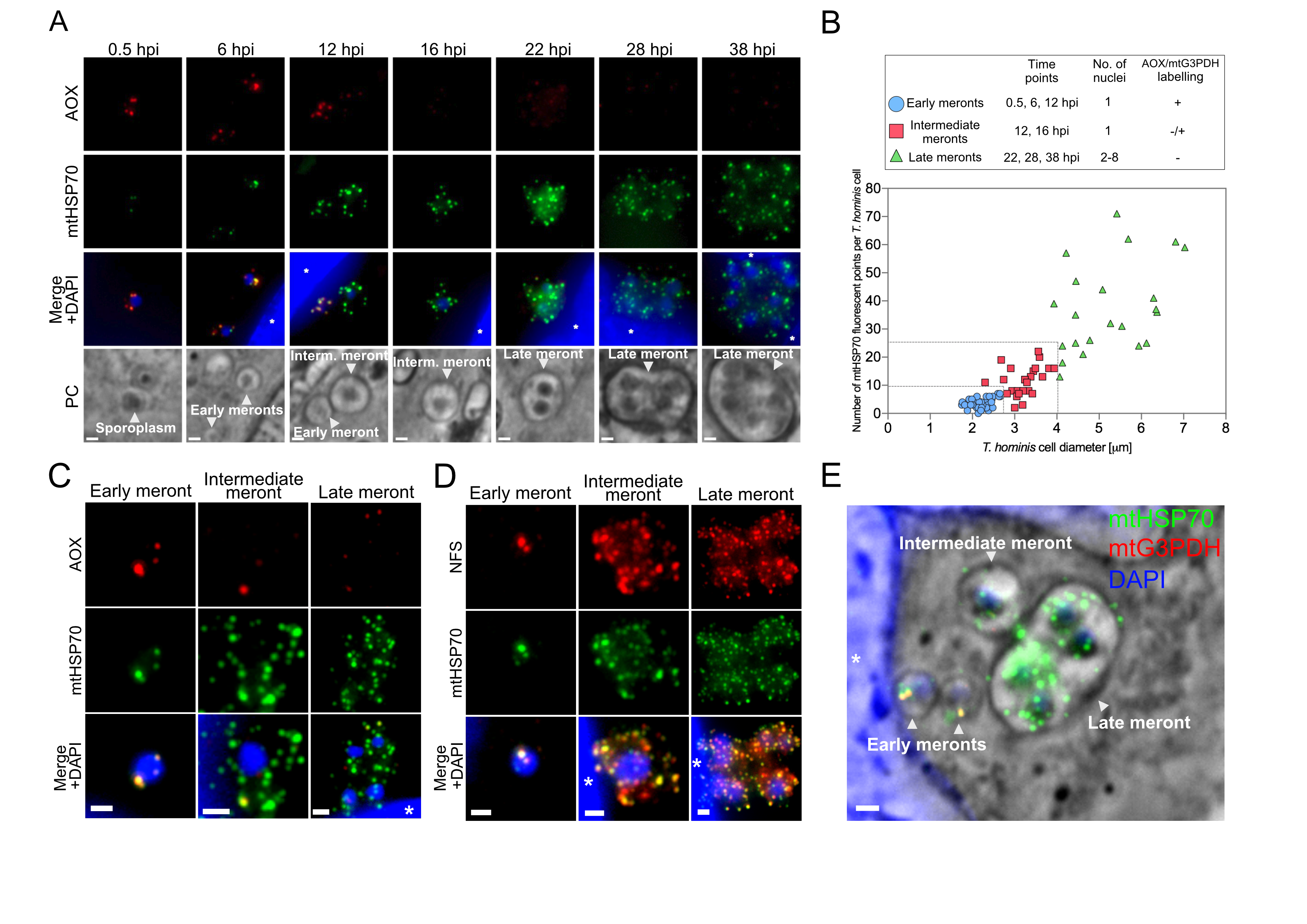

Supplement: S4 Fig — Monolayers of T. hominis infected host cells sampled at different time points after the initiation of the infection (A, C, D), or from a mixed non-synchronised infection (E) were fixed with methanol-acetone and double labelled with the affinity purified polyclonal rabbit antibodies against T. hominis AOX (red, A, C), T. hominis mtG3PDH (red, E), or T. hominis NFS (red, D); and polyclonal rat antisera raised against T. hominis mtHSP70 (green, A-E). (A) Immunofluorescence and phase contrast images of the T. hominis cells observed at different time points across the time course of the synchronised infection. (B) Three distinct morphotypes (top panel) of the parasite cells imaged across the time course of the infection (A) were identified based on: the number of the observed nuclei, and the proportion of the numbers of the quantified mitosomal AOX/mtG3PDH and mtHSP70 fluorescent points. Early meronts; observed mostly at 0.5–6 hpi, and infrequently at 12 hpi; had a single nucleus; cell diameter of 2.25 μm ± 0.29 μm; and all of their mitosomes were double labelled with AOX/mtG3PDH and mtHSP70 antibodies. Intermediate meronts; observed mainly between 12–16 hpi; had a single nucleus; cell diameter of 3.20 μm ± 0.39 μm; and most of their mitosomes were labelled only with mtHSP70/NFS antibodies. Late meronts; observed after 22 hpi; had usually 2 or 4 nuclei; cell diameter of 5.23 μm ± 0.95 μm; and virtually all their mitosomes were labelled only with mtHSP70/NFS antibodies. The fourth morphotype observed at early proliferative stages of infection was a sporoplasm (A and S5 Fig, 0.5 hpi), characterised by a single nucleus; cell diameter generally smaller than that of an early meront; and amorphous shape as opposed to the ovoid shape of the meronts. The sporoplasms most likely corresponded to the contents of the spore injected into the host cell that initiates the infection. Sporoplasms were notoriously difficult to image due to their small size, extremely weak DAPI signal (rel [file ppat.1011024.s004.tiff]

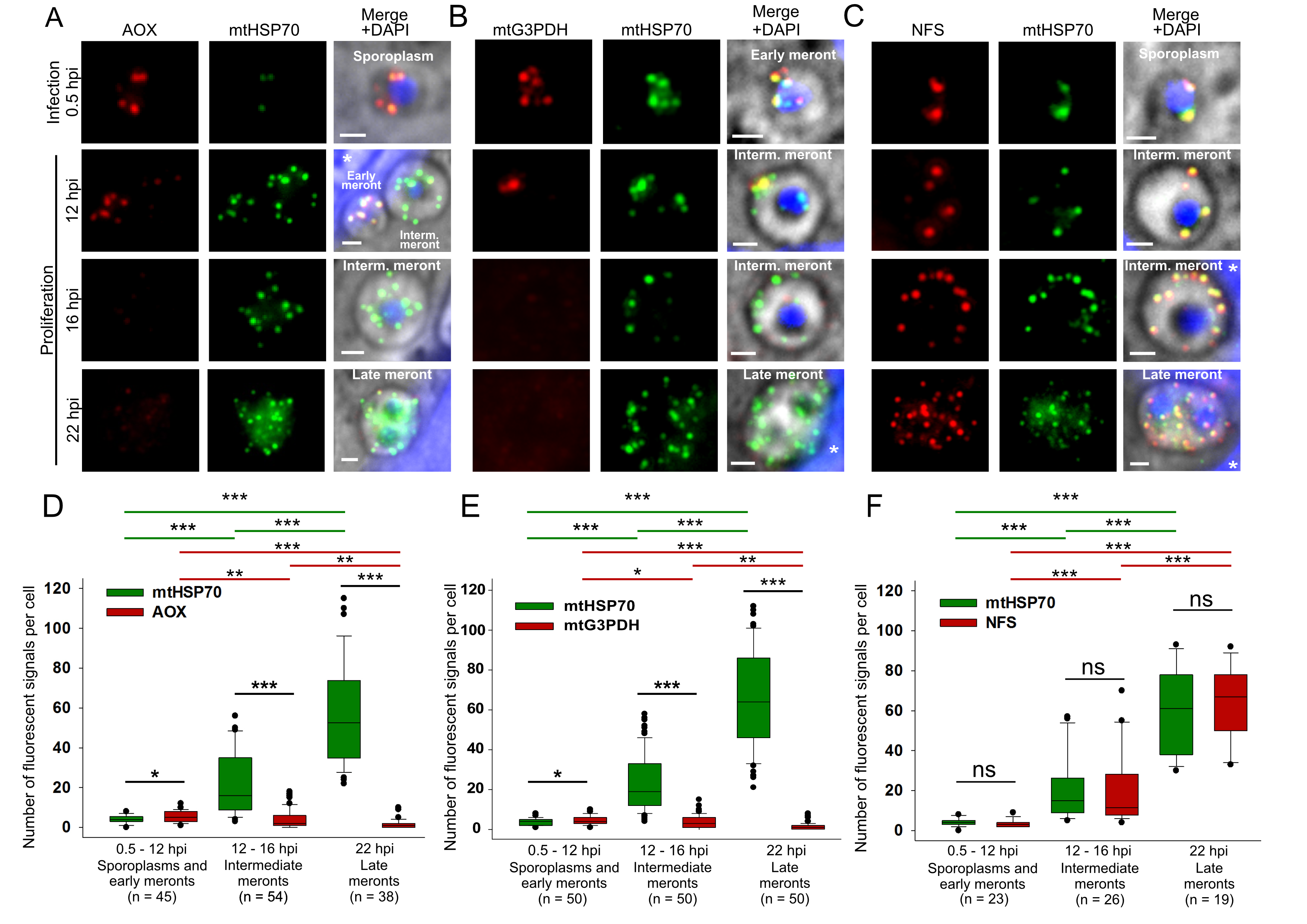

Supplement: S5 Fig — Comparison of the numbers of fluorescent signals detected in different samples, such as different time points from a time course of infection experiments, can be affected by a sample-to-sample variation due to factor such as differences in thickness of the host cell monolayer, or small variations in the sample preparation (e.g. fixation temperature) despite following the same protocol. It can be particularly challenging when trying to compare numbers of signals that display a life-cycle stage specific labelling pattern, such as those detected using anti-AOX and anti-mtG3PDH antibodies. In order to directly compare different stages of the parasite life cycle in a single sample, numbers of fluorescent point signals were quantified (D-F) in randomly sampled fluorescent images of non-synchronised mixed population of T. hominis (S13 Fig). All morphotypes identified in the time course of infection (A-C) were also found in the samples of mixed population (S4E and S13 Figs). Only images that contained at least two out of three major morphotypes (early meronts, intermediate meronts, and late meronts) in a single field of view were selected for the analyses. Average number of the red (AOX) and the green (mtHSP70) fluorescent point signals was measured for a total of 137 cells observed in 20 z-stacks from two independent biological replicates corresponding to two independently infected and cultured populations of T. hominis infected RK13 cells (10 z-stack for each of the replicates). No statistically significant differences were found between the replicates for any of the measurements (T-test). Numbers of the analysed parasite cells (n) were displayed below the plots. The significance (T-test) of the differences in number of the detected fluorescent points was displayed above the plots (*** P ≤ 0.001, ** P ≤ 0.01, * P ≤ 0.05, not significant P > 0.05). (TIFF) [file ppat.1011024.s005.tiff]

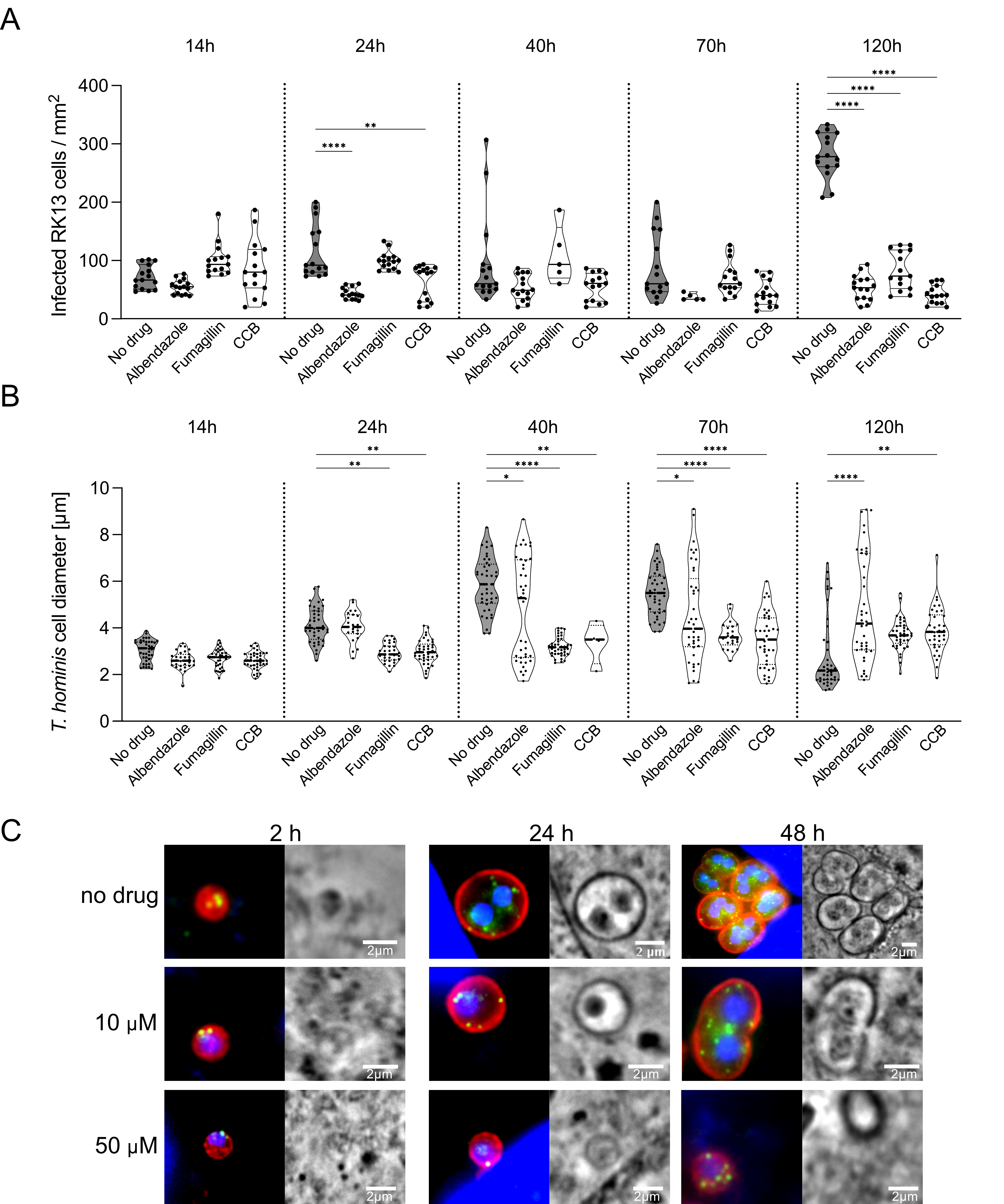

Supplement: S6 Fig — (A, B) Violin plots of the numbers of the T. hominis infected RK cells (A), or the diameter of T. hominis cells (B) observed across a time course of infection in presence of the drugs (albendazole, fumagillin, and CCB), and in the non-treated control (No drug). The displayed data is the same as that in Fig 3 but was presented relative to the non-treated control. Datapoints were displayed as black circles. ANOVA analysis was used to test significance of the differences between the measured values (****, P ≤ 0.0001; ***, P ≤ 0.001; **, P ≤ 0.01; *, P ≤ 0.05). All experiments were performed in three biological replicates. (C) Confocal immunofluorescence images of representative T. hominis cells labelled with rabbit antibodies against ThNTT4 (red) and rat antibodies against mtHSP70 (green); observed in samples collected at 2h, 24h, and 48h time points post infection in samples treated with CCB at concentrations required for either partial (10 μM) or complete (50 μM) inhibition of the parasite life cycle, relative to the non-treated control (no drug). (TIFF) [file ppat.1011024.s006.tiff]

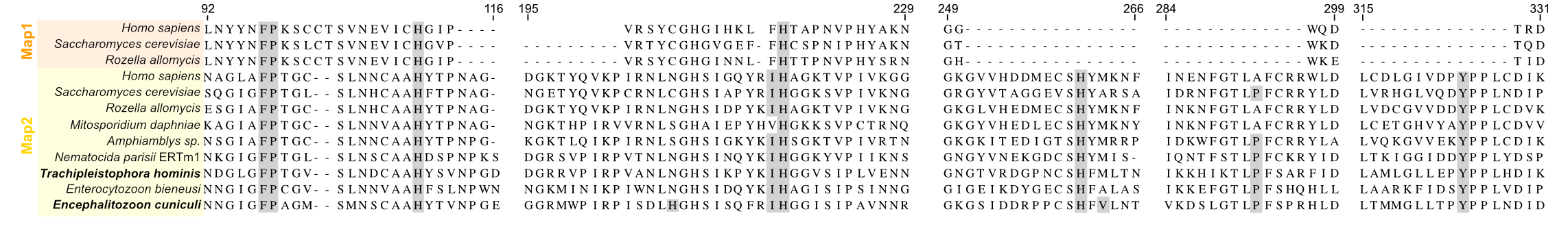

Supplement: S7 Fig — Sequences of Map1 and Map2 from S. cerevisiae, H. sapiens, and R. allomycis; and Map2 from microsporidia were aligned using Muscle [62].The residues contacting the fumagillin and TNP470 in the crystal structure of E. cuniculi Map2 (PDB 3FM3) [39] were indicated (red arrowheads). (TIFF) [file ppat.1011024.s007.tiff]

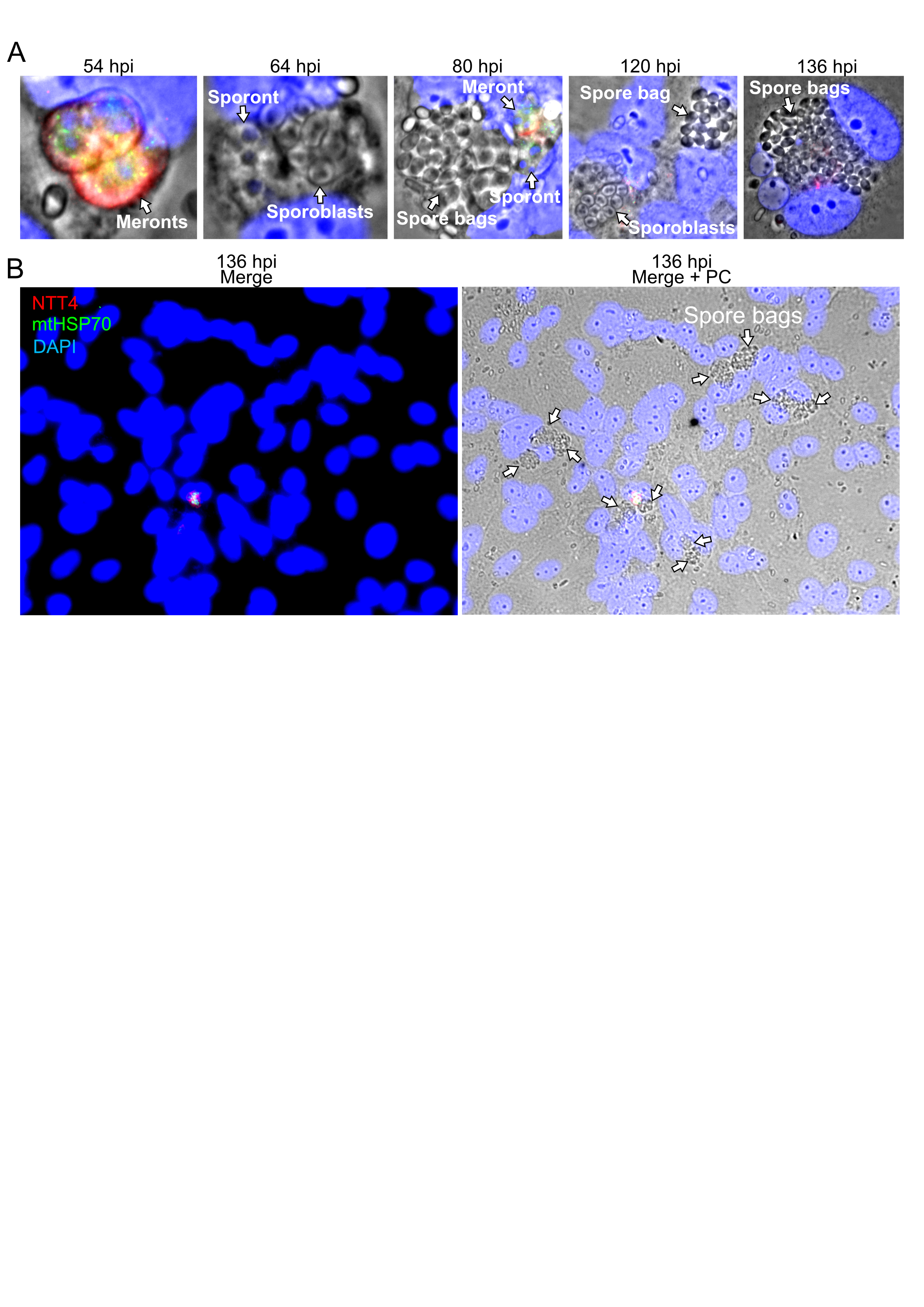

Supplement: S8 Fig — (A) Images of representative T. hominis cells observed in the samples collected across the time course of infection where, colletochlorin B (CCB) was added to the medium 22 hours after the addition of spores to the non-infected host cells, when AOX and mtG3PDH labelled mitosomes were absent from virtually all parasite cells (S5 Fig). Representative cells of the parasite life cycle stages observed at each time point; meronts (54 hpi), sporonts (64–80 hpi), sporoblasts (64–120 hpi), and spores (80–130 hpi); were indicated on the images (white arrows). Time points displayed above the images refer to the time points after the addition of the spores. (B) Low magnification image of the CCB treated samples at 136 hpi. All infected host cells contained spore bags (white arrows), but no new infections were observed. Cells labelled with antibodies represent late meront stages from the initial infection that have not entered the spore formation stage of life cycle. All samples were double-labelled with the antibodies against ThNTT4 (red), and ThmtHSP70 (green). (TIFF) [file ppat.1011024.s008.tiff]

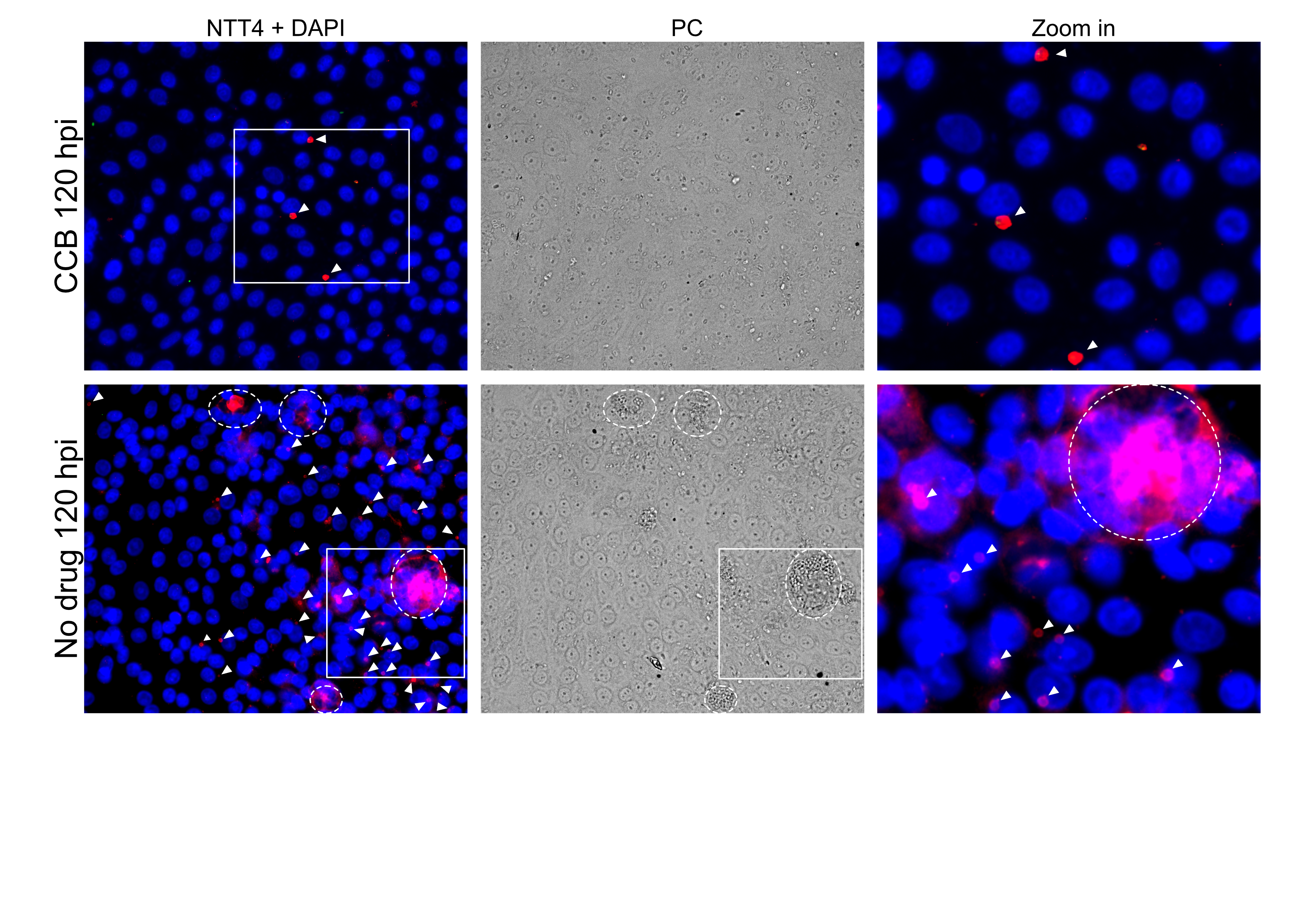

Supplement: S9 Fig — 120 hours after the addition of spores, in the culture of the T. hominis infected RK13 host cells treated with the CCB (top panel), only the persistent small T. hominis meronts (white arrowheads) from the initial infection were observed. At the same time point in the non-treated control (bottom panel), significantly higher number of the small meronts and sporoplasms (white arrowheads) from the secondary infection were observed. Smaller number of the heavily infected host cells from the primary infection (dashed circles); filled with the infective spores, and other stages of the parasite life cycle; were observed alongside the newly infected host cells. The heavily infected RK13 cells were characteristically multi-nucleated, with the host nuclei surrounding the foci of the infection. High intensity of fluorescence observed in the heavily infected cells is due to longer exposure times required to image the sporoplasms labelled with NTT4. The difference in the fluorescence intensity between the sporoplasms and late meronts observed in the same image is likely a result of an accumulation of the NTT4 across the intracellular stage of T. hominis life cycle. NTT4 belongs to the top 10% most abundant transcripts measured across the parasite life cycle [5], and the apparent high levels of NTT4 in the cell membrane of the larger late stage meronts are consistent with its proposed critical role in supporting the rapid parasite growth [5]. All samples were double-labelled with the antibodies against ThNTT4 (red), and ThmtHSP70 (green). Signal in red channel was highly exposed in order to visualize the small meronts and sporoplasms labelled weakly relative to the high intensity of labelling observed for the intermediate and late meronts. (TIFF) [file ppat.1011024.s009.tiff]

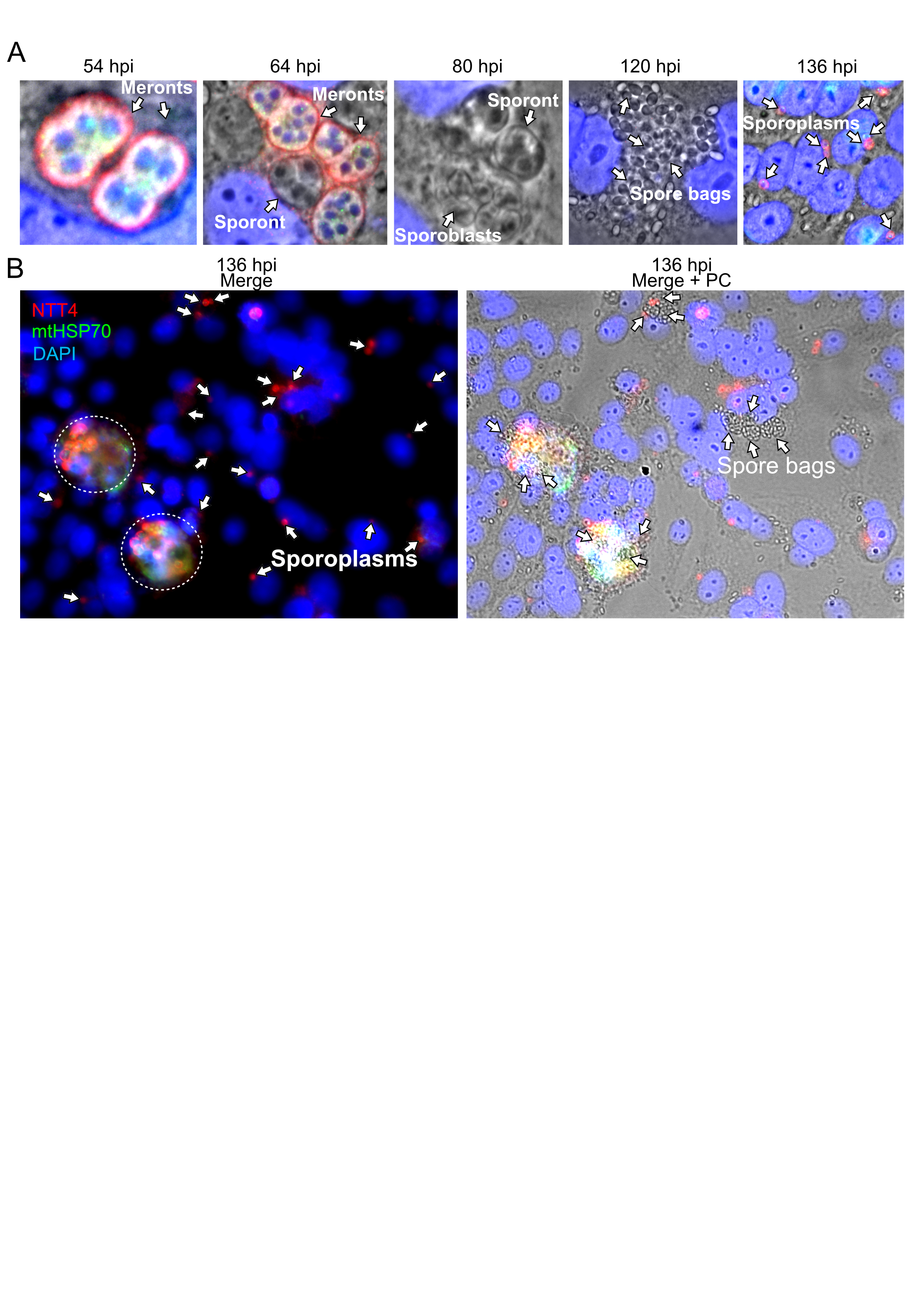

Supplement: S10 Fig — Colletochlorin B (CCB) was added to the culture medium containing T. hominis spores and incubated at 4°C for 16 hours, followed by at least 1 hour of incubation at 37°C prior to the addition of the medium containing spores and the drug to the monolayer of uninfected RK13 cells. 22 hours post infection, medium containing the CCB was replaced with a culture medium without the drug. (A) Images of representative T. hominis cells observed in the samples collected across the time course of infection where, the CCB was removed at 22 hpi. Representative cells of the parasite life cycle stages observed at each time point; meronts (54–136 hpi), sporonts (64–136 hpi), sporoblasts (80–136 hpi), spores (120–136 hpi), and sporoplasms (136 hpi); are indicated on the images (white arrows). Time points displayed above the images refer to the time points after the addition of the spores. (B) Low magnification image of the CCB treated samples at 136 hpi. Heavily infected host cells containing meronts, spore forming stages (sporonts and sporoblasts), and spores are indicated with the dashed circles. White arrows indicate sporoplasms inside newly infected host cells (Merge), or spore bags inside the host cells infected during the primary infection (Merge + PC). All samples were double-labelled with the antibodies against ThNTT4 (red), and ThmtHSP70 (green). (TIFF) [file ppat.1011024.s010.tiff]

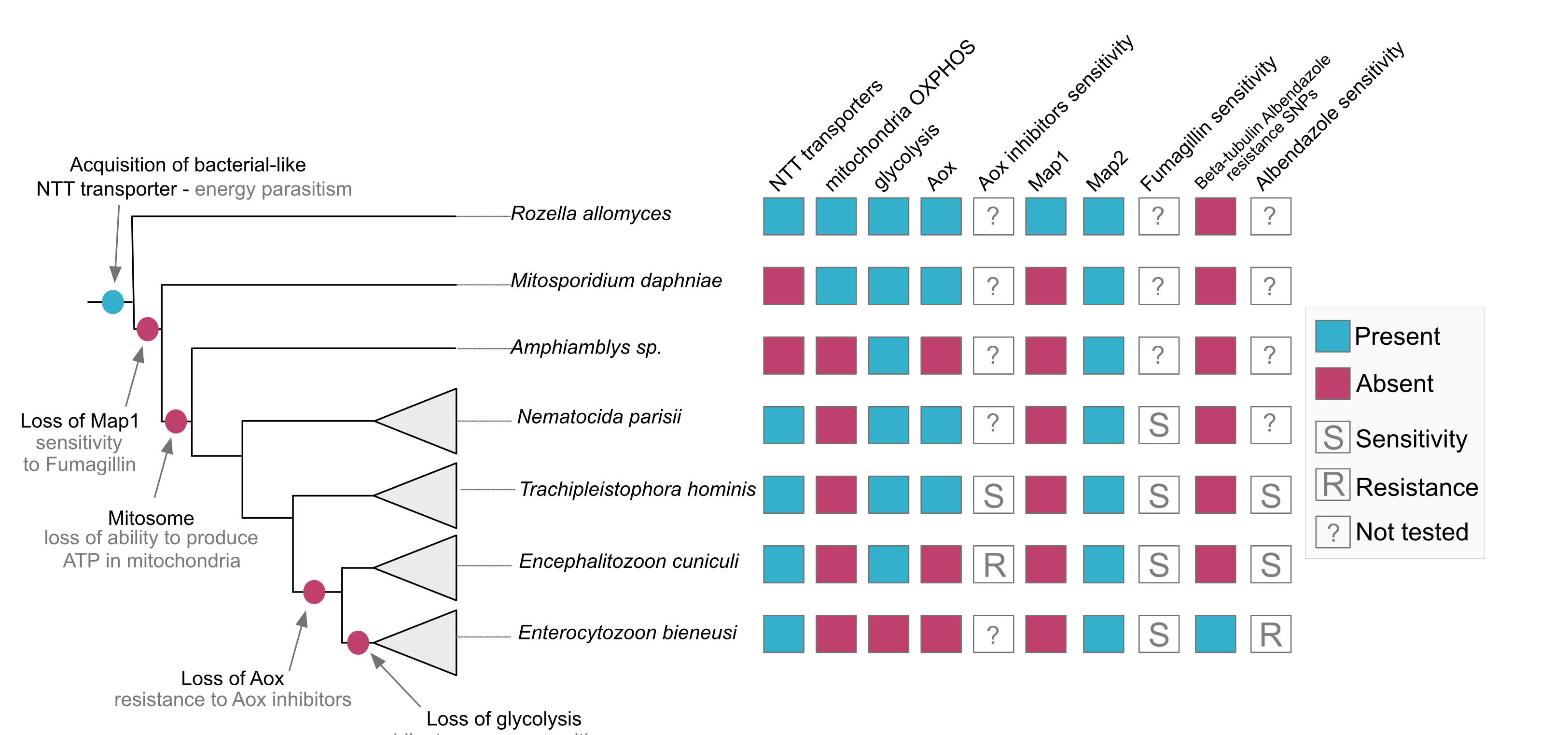

Supplement: S11 Fig — Inferred gene gain (Fig 2A) of the ancestral nucleotide NTT transporter (turquoise), as well as inferred gene losses (Fig 2A) of key pathways for energy generations (magenta) were indicated on the cladogram of microsporidia. In the table, coloured squares indicated presence (blue) or absence (red) of genes and pathways for energy generation in genomes of microsporidia and R. allomyces. Presence or absence of certain genes, or amino acid residues in protein sequences, can be associated with sensitivity to antimicrobial agents in microsporidia. Sensitivity to the AOX inhibitor CCB is associated with presence of the AOX gene (this study). Sensitivity to the fumagillin is likely to be due to the absence of the Map1 gene [39] from the microsporidian genomes (S7 Fig), and the sensitivity to the albendazole is associated with a presence of a specific residues in Beta-tubulin amino acid sequence [63,64] (S16 Fig). (TIFF) [file ppat.1011024.s011.tiff]

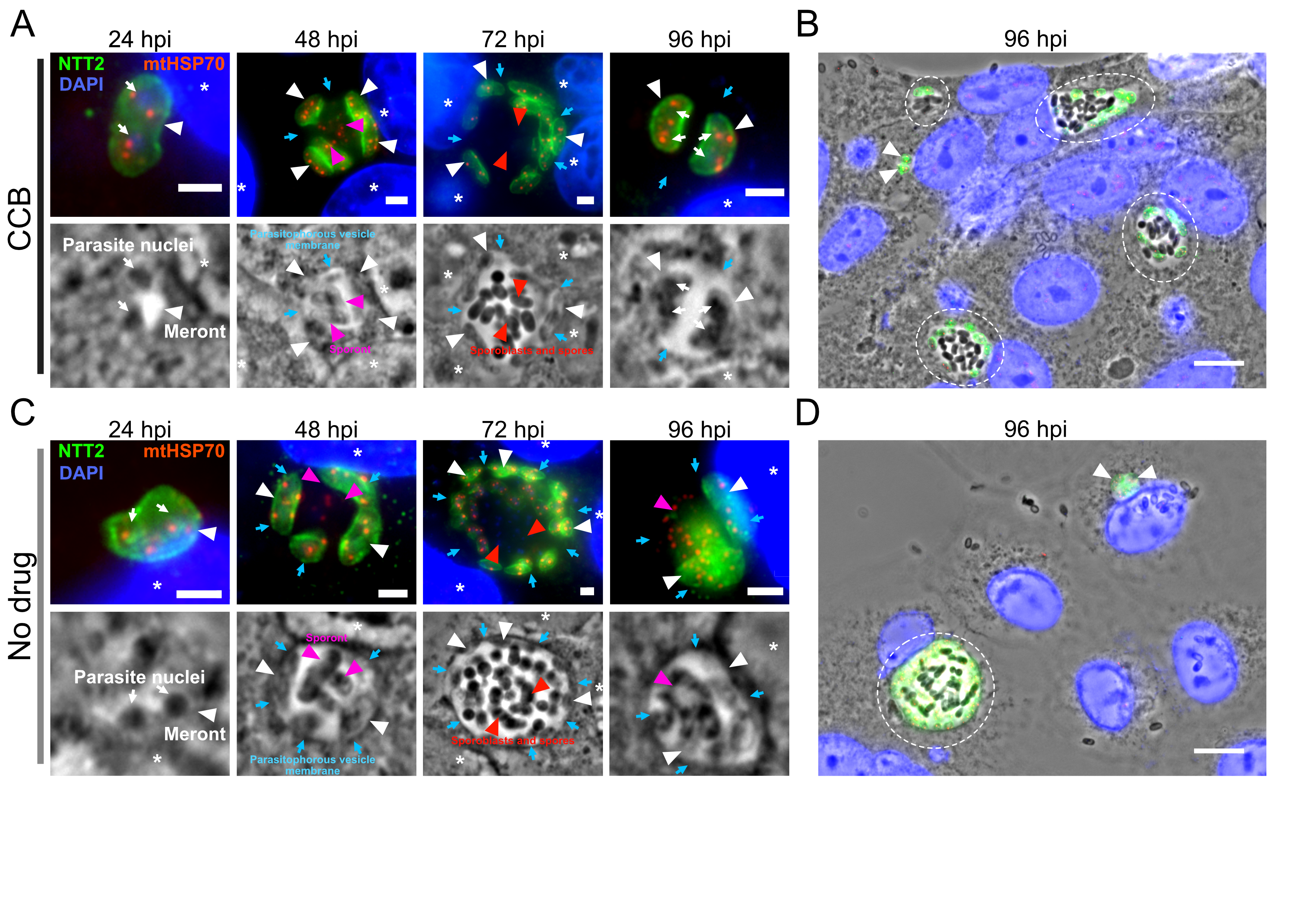

Supplement: S12 Fig — (A, C) As opposed to the T. hominis infection, where no clear organisation of the infection foci can be observed, E. culiculi develops inside the parasitophorous vesicle (turquoise arrows) throughout its life cycle, with a clear stratification of the different life cycle stages across the vesicle. Meronts (white arrowheads) are always observed at the edge of the vesicle; and are strongly labelled with the anti-EcNTT2 antibodies (green), and anti-EcmtHSP70 antibodies (red). Deeper inside the vesicles sporonts (magenta arrowheads) are clearly visible in the phase contrast images, and their mitosomes are labelled with the anti-EcmtHSP70 antibodies, but no labelling with the anti-EcNTT2 antibodies can be observed. The sporoblasts and spores (red arrowheads) are not labelled with either EcmtHSP70 or EcNTT2 antibodies. The fluorescence images in the panels A and C are the same as in the Fig 4. (B, D) Low magnification images of the CCB-treated (B) or non-treated (D) E. cuniculi-infected RK13 culture at 96 hpi. E. cuniculi meronts, spore forming stages, and newly formed spores were observed inside the host cells infected during the primary infection (dashed circles). The host cells infected during the secondary infection by the newly formed spores contained only the meronts (white arrowheads). The white arrows indicated E. cuniculi nuclei which were often more clearly visible in the phase contrast images than in the DAPI-labelled (blue) fluorescence images, due to the weak intensity labelling of small E. cuniculi nuclei relative to the high intensity DAPI-labelling of the host nuclei (asterisk). (TIFF) [file ppat.1011024.s012.tiff]

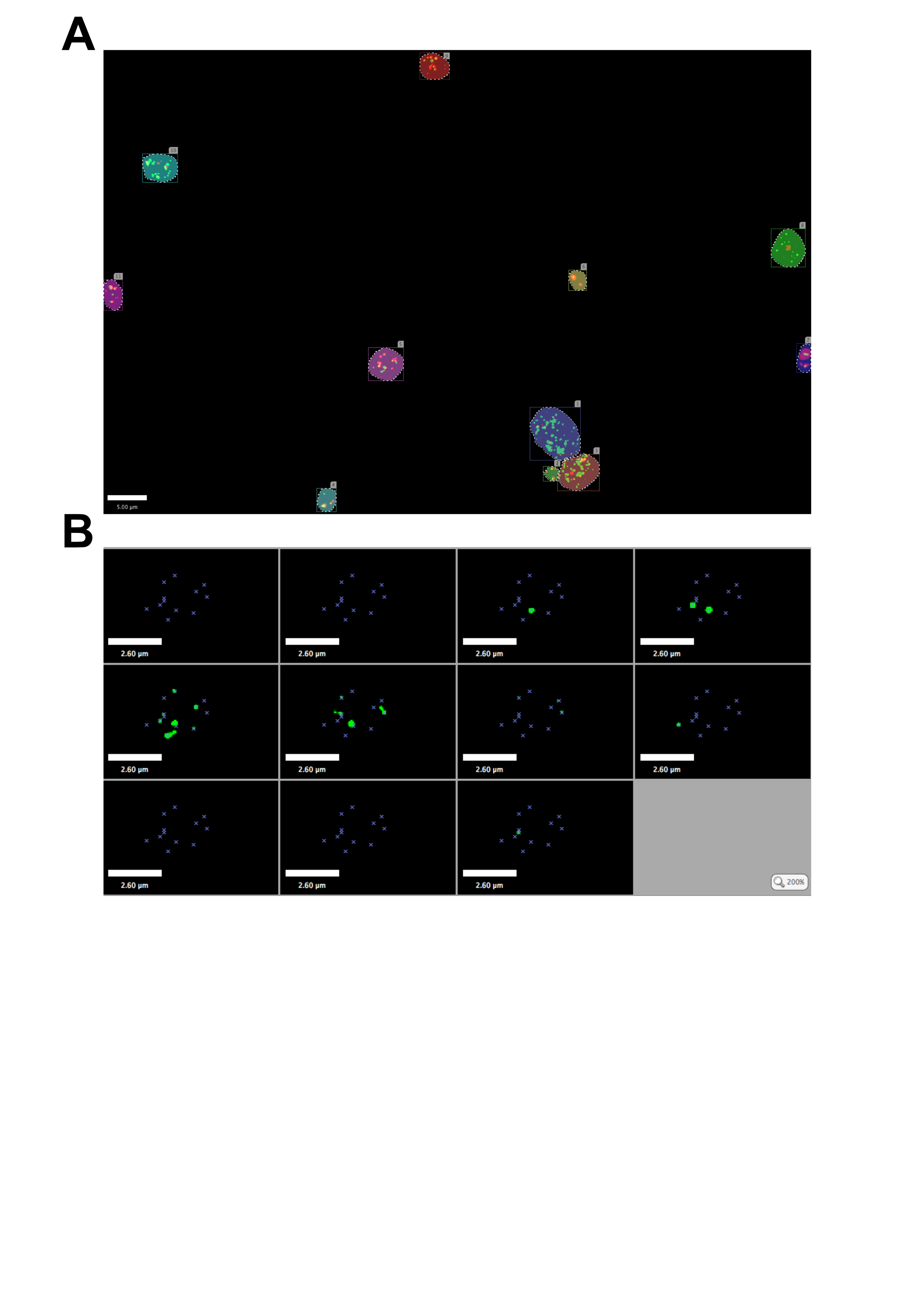

Supplement: S13 Fig — (A) Z-stack of a representative field of view used for the quantification of the fluorescent points (Figs 1F and S5) detected with the affinity purified antibodies against ThAOX (red) and polyclonal sera against ThmtHSP70 (green). The images in the red and the green channel were highly overexposed in order to visualize the lowest intensity points. The exposure times and the thresholds were set for all samples so that no signals were detected in the controls prepared the same way as the analysed samples but without the primary antibodies, and so that the highest intensity points were not overlapping. Dashed lines indicate the areas of single cells identified based on the phase contrast image. (B) Representative images of the T. hominis mitosomes labelled with the ThmtHSP70 (green) antibodies, sampled across the parasite cell in the Z-axis at the optimum sampling distance automatically set using the Apotome.2 ZEN software (Zeiss). Blue Xs indicate position of the fluorescent points detected using Volocity’s (Perkin Elmer) ‘local intensity maxima’ detection algorithm (‘Find spots’ function). Thresholds were adjusted so that only points which were not overlapping, were detected in all cells within a single field of view. Widefield fluorescence microscopy (WFM) was selected over laser confocal scanning microscopy (LCSM) based on a number of factors, mainly: no observed photobleaching; good signal to noise ratio; and the ability to image all parasite cells in phase contrast, which was crucial for the rigorous classification of the different morphotypes of the parasite cells. The photobleaching using the LCSM was especially high in many parasite cells labelled with the anti-ThAOX and the anti-ThmtG3PDH antibodies, likely due to the low abundance of these mitosomal proteins, which is consistent with the low intensity of the western-blot bands detected using the same antibodies against the protein extracts from the samples enriched in intracellular stages of the parasite (Fi [file ppat.1011024.s013.tiff]

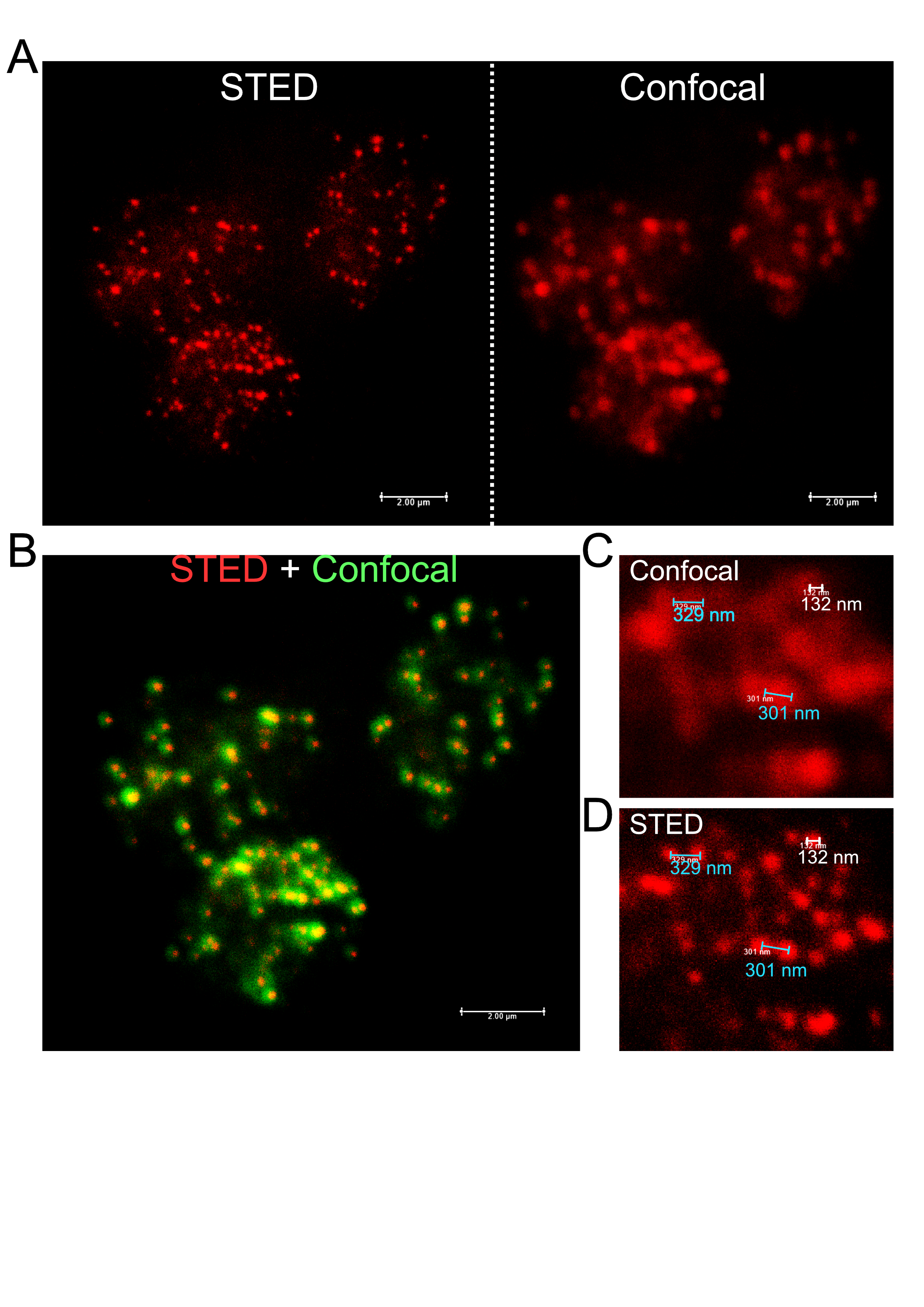

Supplement: S14 Fig — (A) Single confocal section of two adjacent late T. hominis meronts imaged using regular laser confocal scanning microscopy (LCSM, right panel), and super resolution STED (left panel) modes of Leica SP8 STED microscope. (B) Overlay of the STED (red), and the regular LCSM (green) images from (A). (C, D) Measurements of the diameter of a single florescent point (white line), or of the distance between the centres of two fluorescent points (turquoise line), measured in the STED image (D). The higher resolution of the STED often resolves a single fluorescent point observed in the confocal into two adjacent points. The STED microscopy also allows imaging of the mitosomes at the resolution closer to their size measured in electron microscopy images (∼50 nm x 90 nm) [10]. Despite at least two times lower resolution of the diffraction limited microscopy (regular LSCM, and wiedefield microscopy) the results obtained using these methods provided a good approximation for the analyses of variation in the numbers of the mitosomes in the different stages of the parasite life cycle observed in this study. (TIFF) [file ppat.1011024.s014.tiff]

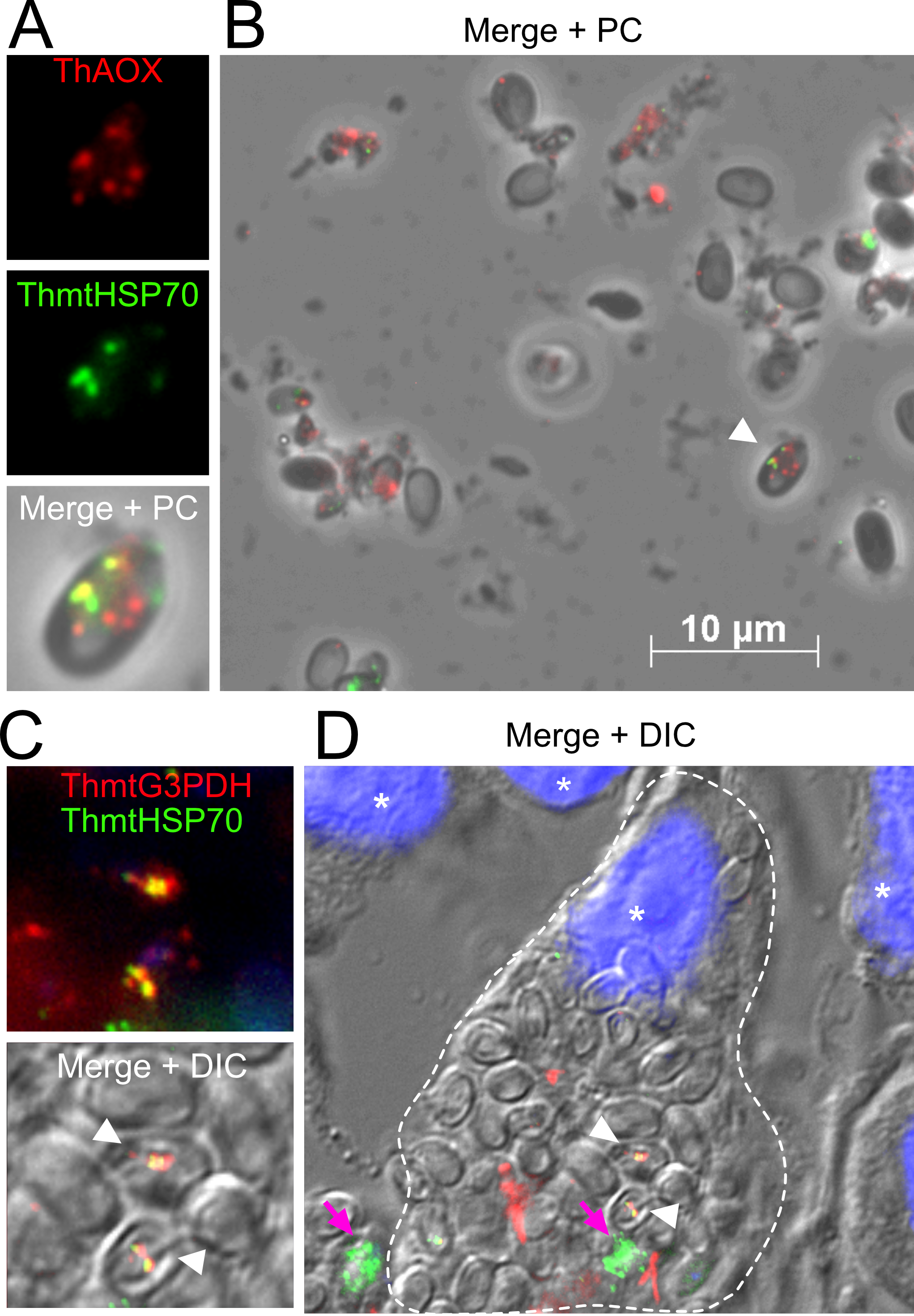

Supplement: S15 Fig — Due to the difficulty in penetrating the microsporidian spore wall with the antibodies in samples fixed using either Methanol-Acetone or PFA-detergent fixation-permeabilization methods, in effort to label the interior of the spores we decided to use thin cryo-sections of the spores purified from the medium collected from the culture of T. hominis infected RK13 cells monolayer (A and B), as well as the cryo-sections of the T. hominis infected RK13 monolayer (C and D). The spores were purified using Percol gradient, and the spore pellets were suspended in the O.C.T. (optimal cutting temperature) compound and frozen in liquid nitrogen. The monolayers were, washed with PBS, scraped off the culture flask surface using a cell scraper, and suspended in the OCT. 3 μm cryo-sections of the samples were cut using cryo-microtome (Leica), placed on microscopy slides, and processed according to the standard immunofluorescence protocol (Material and Methods). (A) Environmental (i.e. collected from the medium covering the infected host cells) spores labelled with the affinity purified antibodies against ThAOX (red), and ThmtHSP70 (green), and imaged in phase contrast (PC); were found in the sectioned samples of the purified spores. (B) The majority of the spores were not labelled with the antibodies suggesting a low efficiency of sectioning. Furthermore, debris were observed throughout the sample. The debris were often labelled with the specific antibodies suggesting they may be cellular debris from the germinated and/or damaged spores. The spore apparently labelled with the specific antibodies (A) was annotated with the white arrowhead. (C) Intracellular spores labelled with the affinity purified antibodies against Th mtG3PDH (red), and ThmtHSP70 (green) and imaged using the differential interference contras (DIC) observed inside the host cell (white arrowheads). (D) Heavily infected host cell filled with the T. hominis spores was outlined with the dashed line. A single meront (ma [file ppat.1011024.s015.tiff]

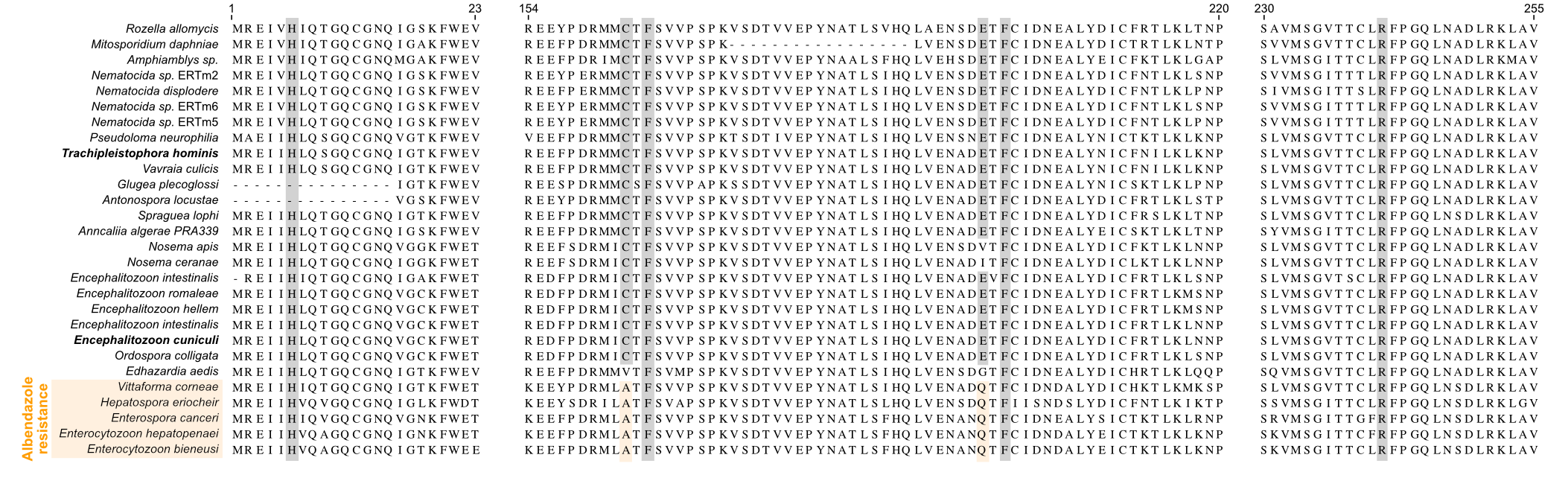

Supplement: S16 Fig — Amino acid sequences of microsporidian Beta-tubulin were aligned using Muscle [62]. The residues reported to be associated with the benzimidazole sensitivity [63,64] were indicated (red arrowheads). (TIFF) [file ppat.1011024.s016.tiff]
